# Stress-responsive pathways and small RNA changes distinguish variable developmental phenotypes caused by MSH1 loss

**DOI:** 10.1186/s12870-017-0996-4

**Published:** 2017-02-20

**Authors:** Mon-Ray Shao, Sunil Kumar Kenchanmane Raju, John D. Laurie, Robersy Sanchez, Sally A. Mackenzie

**Affiliations:** 1 Department of Agronomy and Horticulture, University of Nebraska-Lincoln, Lincoln, NE USA; 2 Sainsbury Laboratory, University of Cambridge, Cambridge, UK

**Keywords:** MSH1, Transcriptome, Plastid, Organelle, Stress, Small RNA

## Abstract

**Background:**

Proper regulation of nuclear-encoded, organelle-targeted genes is crucial for plastid and mitochondrial function. Among these genes, *MutS Homolog 1* (*MSH1*) is notable for generating an assortment of mutant phenotypes with varying degrees of penetrance and pleiotropy. Stronger phenotypes have been connected to stress tolerance and epigenetic changes, and in *Arabidopsis* T-DNA mutants, two generations of homozygosity with the *msh1* insertion are required before severe phenotypes begin to emerge. These observations prompted us to examine how *msh1* mutants contrast according to generation and phenotype by profiling their respective transcriptomes and small RNA populations.

**Results:**

Using RNA-seq, we analyze pathways that are associated with *MSH1* loss, including abiotic stresses such as cold response, pathogen defense and immune response, salicylic acid, MAPK signaling, and circadian rhythm. Subtle redox and environment-responsive changes also begin in the first generation, in the absence of strong phenotypes. Using small RNA-seq we further identify miRNA changes, and uncover siRNA trends that indicate modifications at the chromatin organization level. In all cases, the magnitude of changes among protein-coding genes, transposable elements, and small RNAs increases according to generation and phenotypic severity.

**Conclusion:**

Loss of *MSH1* is sufficient to cause large-scale regulatory changes in pathways that have been individually linked to one another, but rarely described all together within a single mutant background. This study enforces the recognition of organelles as critical integrators of both internal and external cues, and highlights the relationship between organelle and nuclear regulation in fundamental aspects of plant development and stress signaling. Our findings also encourage further investigation into potential connections between organelle state and genome regulation vis-á-vis small RNA feedback.

**Electronic supplementary material:**

The online version of this article (doi:10.1186/s12870-017-0996-4) contains supplementary material, which is available to authorized users.

## Background

As sessile organisms, plants must be able to perceive and adapt to changing environmental cues such as stress conditions. Stresses can be present in combinations, such as the simultaneous heat and drought, or a combination of abiotic and biotic stresses, and can be highly detrimental to plant growth [1, 2]. Under such circumstances, plant responses are often non-additive compared to the individual stresses, suggesting a complex regulatory network with significant crosstalk [2, 3]. Understanding these regulatory networks, their signaling components, and the genes that influence them are therefore important topics in plant biology.

Because plastids are required for photosynthesis, the production of major phytohormones, and other metabolic processes, they are in a key position to sense alterations in the environment and communicate accordingly to the nucleus [4]. For example, light quality affects the plastid transcriptome while light quantity affects plastid signaling, and both influence photomorphogenesis [5, 6]. Plastids also play a role in tolerance and signaling against drought, freezing, heat, and oxidative stress [4, 7, 8]. Furthermore, disruption of regulators of plastid function, such as pentatricopeptide repeat (PPR) proteins, can lead to defects in growth, embryo development, photosynthesis, and leaf pigmentation, among others [9]. As such, the status of plastids has a profound level of control over the entire plant, including its responses to environmental and cellular stress.

*MSH1*, previously known as *CHM1*, is a plant-specific, nuclear-encoded *MutS* homolog that is targeted to both plastids and mitochondria. Loss of *MSH1* causes an array of phenotypes, including variegation, dwarfism, altered leaf morphology, delayed flowering, and male sterility [10–13]. Additional phenotypes are environmentally-dependent, such as secondary stem growth and aerial rosette formation under short-day conditions [14]. From *MSH1*-suppressed RNAi lines in sorghum, pearl millet, tomato, tobacco, and soybean, it is apparent that many of these phenotypes are conserved between monocots and eudicots [14–17]. In addition, manipulation of *MSH1* is associated with tolerance to heat, high light, and drought [15, 18, 19], particularly in *MSH1*-depleted plants showing strong developmental phenotypes. Indeed, *MSH1* transcript levels are endogenously down-regulated during stress [15], leading to the possibility that in *msh1* mutants, stress responses are triggered to cause growth suppression and other phenotypes.

A further consequence of *MSH1* loss is epigenetic, with evidence first appearing from the segregation of *MSH1* RNAi plants maintained for multiple generations as hemizygotes. A proportion of subsequent wild-type segregant progeny lacking the RNAi transgene still retained altered growth and delayed flowering phenotypes, which were not cytoplasmically heritable [14]. Furthermore, whole genome bisulfite-sequencing of *msh1* T-DNA mutants revealed numerous changes in DNA methylation over both gene bodies and transposable elements [20]. In plants, one role of DNA methylation is used to silence transposable elements, which can become activated in during stress conditions [21]. In some cases, changes in DNA methylation have also been associated with stress-induced gene regulation, such as during phosphate starvation or *Pseudomonas syringae* infection [22, 23], and may also provide the mechanisms basis for stress priming and memory [23, 24].

During propagation of the *msh1* T-DNA materials, we observed that first-generation homozygous *msh1* mutants (S1) had either no phenotype or only slight variegation, whereas second-generation homozygous *msh1* mutant (S2) plants displayed the full range of *msh1*-associated phenotypes [20]. Compared to S1 generation *msh1* plants, S2 generation *msh1* plants with mutant phenotypes also had markedly increased amounts of methylation changes in the non-CG context [20]. This contrast raises questions as to what transcriptional changes begin to occur in the *msh1 -/-* S1 plants, as opposed to *msh1* -/- S2 plants or later generations. We hypothesized that the degree of gene expression changes would parallel phenotype and methylome state, distinguishing the transition between the S1 and S2 generations. In this study, we performed RNA-seq to identify genes that are altered in the first sporophytic generation of *MSH1* loss, as well as those that are induced with the onset of strong phenotypes in the subsequent generation. Using small RNA-seq, we also show that miRNA profiles and repeat-associated siRNA levels change according to *msh1* generation and phenotype. Together, these data indicate that the variable phenotypes resulting from *msh1* loss are caused by the triggering of large gene expression networks associated with stress and other pathways, which also ultimately influence genome-wide changes in chromatin organization.

## Results

### Phenotypic and transcriptomic changes from MSH1 loss build over two generations

**Fig. 1:**
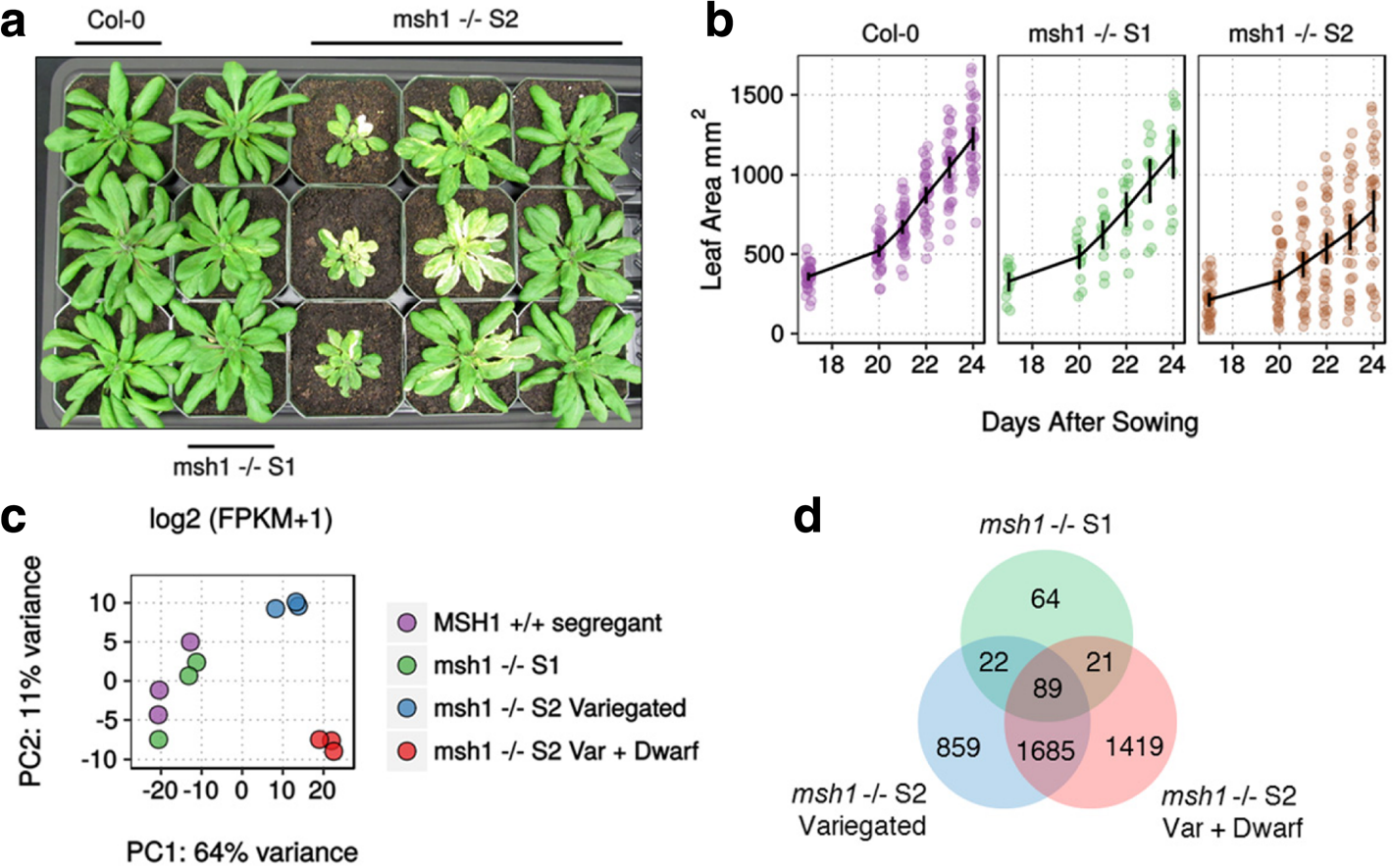
Phenotypic severity is associated with levels of global transcriptome changes in *msh1* T-DNA mutants. **a** Representative images of wild-type Col-0, *msh1* -/- S1 generation mutants, and *msh1* -/- S2 generation mutants. **b** Leaf area with means and 95% confidence limits for wild-type Col-0 (*n* = 204), *msh1 -/-* S1 (*n* = 72), and *msh1* -/- S2 plants (*n* = 186). **c** PCA plot of principal components 1 and 2 based on log2(FPKM + 1) values. **d** Venn diagram of differentially expressed genes in *msh1* mutants compared to *MSH1* +/+ wild-type segregants

We propagated a T-DNA insertion line for the *MSH1* locus that contained a mixture of seeds hemizygous and homozygous for the exon insertion (Additional file 1: Figure S1A-B). By self-pollinating a hemizygous *MSH1* +/- plant, we could observe the phenotype and changes occurring in the immediate progeny generation (S1) lacking *MSH1*. As noted in a previous study [20], these *msh1* -/- S1 plants were mild in phenotype (Fig. 1a), with occasional, slight leaf variegation being the only phenotype observed under normal growth conditions. In contrast, homozygous *msh1* -/- plants from generation S2 show the range of phenotype seen in *chm1-1* mutants, such as strong variegation and a wide variation in leaf area (Fig. 1a-b), growth rate and flowering time. From two S2 lines, variegation was present in approximately 70% of plants (Additional file 1: Figure S1C). As such, mutants showing dwarfism or stunted growth typically, though not necessarily always, also display some form of variegation or chlorotic phenotype.

To determine what transcriptome changes were associated with particular phenotypes, we performed RNA-seq on *msh1* -/- S1 plants, *msh1* -/- S2 variegated plants, and *msh1* -/- S2 variegated & dwarf plants chosen from a single lineage. *MSH1* +/+ wild-type segregants also derived from the hemizygous T-DNA plants were used as the control group. Although there is a possibility that these wild-type segregants may contain subtle dosage-related effects from the previous hemizygous generation, they have no apparent phenotype. Furthermore, they are very closely related in terms of lineage to the *msh1* mutant individuals, an important advantage for the purposes of this study where small RNA changes are also of interest and epigenetics changes may be present [20].

Because of the large overall number of gene expression changes observed in *msh1* mutants, and to minimize the number of potential false positives, we retained only differentially expressed genes (DEGs) identified by both Cuffdiff2 and DESeq2 (see Methods). Compared against the *MSH1* +/+ wild-type segregant control, we found 196 DEGs in *msh1* S1 plants, 2655 DEGs in *msh1* S2 variegated plants, and 3214 DEGs in *msh1* S2 variegated & dwarf plants (Additional file 2), suggesting that increasing phenotype severity is associated with increased gene expression changes. From principal component analysis, the *msh1* S2 groups are also more distinct from the wild-type segregants compared to the *msh1* S1 group (Fig. 1c), and a majority (66%) of the DEGs in *msh1* S2 variegated plants are also shared with *msh1* S2 variegated & dwarf plants (Fig. 1d).

Despite the relatively small total number of DEGs in *msh1* S1 plants, over two-thirds of these are also differentially expressed in at least one of the *msh1* S2 groups. Furthermore, of the 89 genes that are differentially expressed in all *msh1* mutants (S1, S2 variegated, and S2 variegated & dwarf), 38 genes show an intensifying trend between the S1 and S2 generation (Additional file 3: Table S1), defined by at least a four-fold change. Among these, several are transcription factors, such as *SCARECROW-LIKE 13*, *STZ*, *NAC036*, and *DEAR3*. Many are also defense-related, including *WRKY40*, *WRR4*, *LURP1*, *PUB23*, *SDR3*, *CAF1a*, and *CNI1* while others are responsive to environmental conditions such as cold (*CCR1*) or light (*PRIN2*). Therefore, despite the lack of a dramatic outward phenotype, several key pathways already appear to be modulated in the *msh1* -/- S1 plants. On the other hand, 7 genes showed a reversal in differential expression direction (i.e., down-regulation in S1 and up-regulation in S2, or vice versa), including *AOX1D*, the low carbon-induced *PHI-1*/*EXL1*, senescence-associated *SAG13*, the chloroplast-targeted aldo-keto reductase *AKR4C9*, the calcium transporter *CAX3*, and a cold acclimation *WCOR413*-family gene.

An increasing number of studies also indicate that alternative splicing is an important form of regulation in plant development and environmental responses [25, 26]. From our transcriptome data, a gene was considered alternatively spliced if it was identified by Cuffdiff2/spliceR [27, 28] as having differential isoform expression and by JunctionSeq [29] as having differential exon or splice junction usage. Using this methodology, there were 30, 139, and 470 genes with differential isoform expression in the *msh1* S1, *msh1* S2 variegated, and *msh1* S2 variegated & dwarf plants, respectively (Additional file 4). The majority of isoform-specific events pertained to alternative 5′ or 3′ splice sites, followed closely by alternative transcription start or termination sites (Additional file 5: Figure S2D). Roughly half of the isoforms (43 to 54%, depending on sample group) that are differentially expressed in the *msh1* mutants are also predicted to be sensitive to nonsense-mediated decay (Additional file 5: Figure S2E), suggesting that these alternative isoforms may be a means to regulate functional transcript abundance [30]. Nevertheless, similar to gene expression, there is a trend of subtler isoform-specific changes in the *msh1* S1 generation followed by greater changes in the *msh1* S2 generation.

### Expansion of differential expression and splicing in specific pathways reflect upon phenotype

**Fig. 2:**
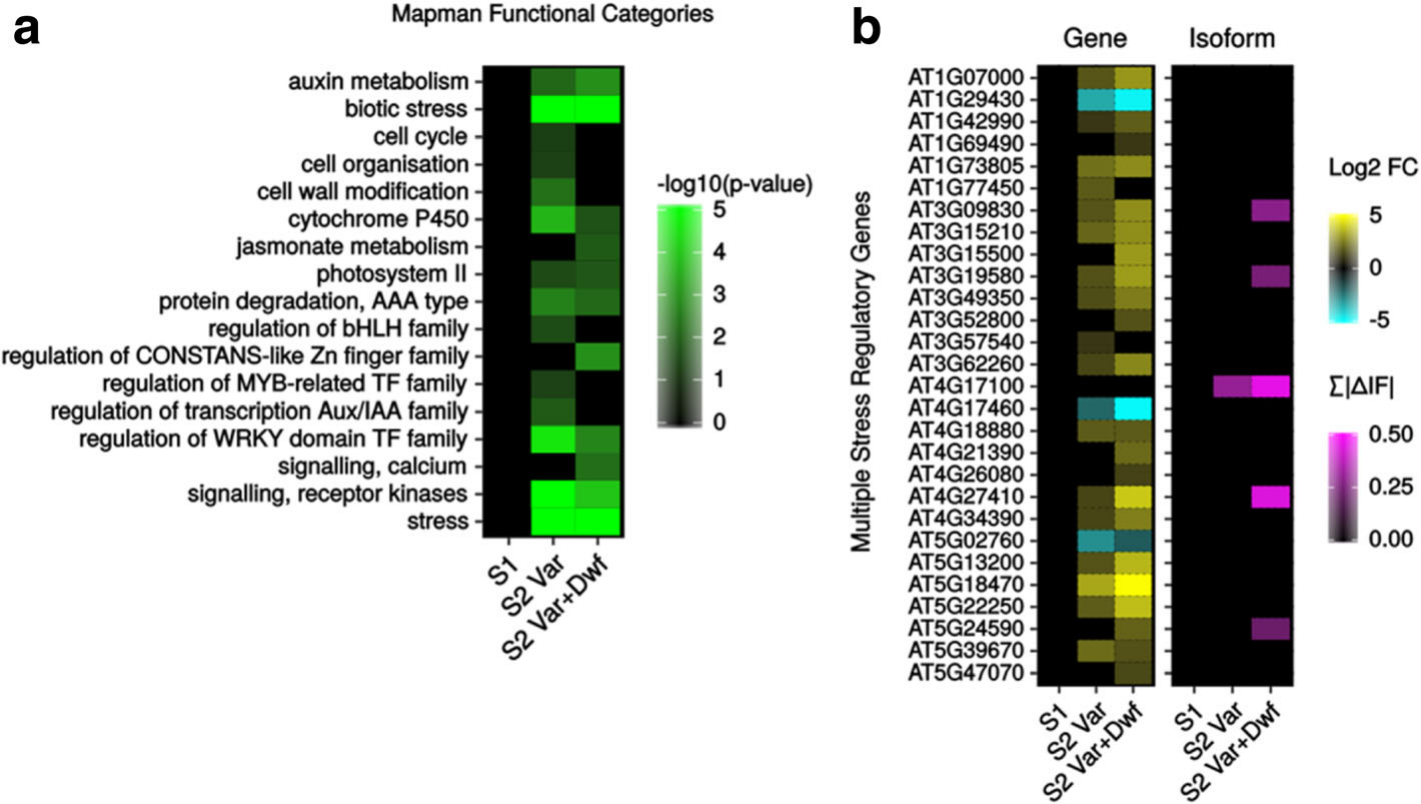
*msh1* S2 mutants share enriched functional categories and changes in general stress gene. **a** Enrichment of MapMan functional categories among differentially expressed genes. **b** Changes in expression of transcripts and isoforms in multiple stress regulatory genes. Gene-level changes are measured by log2 fold-change, while isoform-level changes are measured by the total change in isoform fractions for each gene

We performed enrichment analysis using MapMan [31] functional categories on the differentially expressed genes. Several categories were shared between the *msh1* S2 mutants, such as auxin metabolism, stress, photosystem II components, regulation of WRKY transcription factors, and receptor kinase signaling (Fig. 2a). Cell organization, cell wall modifications, and regulation of bHLH, MYB-related, and Aux/IAA transcription factor families were particularly enriched in the *msh1* S2 variegated plants, while jasmonate metabolism, calcium signaling, and regulation of *CONSTANS*-like transcription factor families were particularly enriched in the *msh1* S2 variegated and dwarf plants. *CONSTANS*-like transcription factors are involved in photoperiod, flowering, and circadian rhythm [32, 33], which may be directly related with delayed growth and development in the dwarfed plants.

Among genes with differential isoform expression in *msh1* S2 variegated & dwarf plants, gene ontology (GO) enrichment analysis [34] found 26 categories that were enriched, including *response to salt stress* and *response to fructose* (Additional file 6). No such enrichment was statistically detectable in *msh1* S1 plants or *msh1* S2 variegated plants, although this is likely affected by the lower overall number of differentially regulated isoforms in those groups. However, nearly all (22/26) of the enriched GO categories from analysis of differentially regulated isoforms in *msh1* S2 variegated & dwarf plants were also found in GO enrichment analysis of differentially expressed genes in *msh1* S2 mutants (Additional file 5: Figure S2F, red arrow), including *MAPK cascade*, *regulation of defense response*, *response to ethylene*, and *response to cold*. Therefore, in addition to differential overall gene expression, alternative isoform regulation and differential splicing likely play a meaningful role in the organismal response to *MSH1* loss.

Together, multiple enrichment analyses indicate that abiotic stress and several related pathways are significantly affected in *msh1* S2 mutants of both phenotypic classes. Among 41 multiple stress regulatory genes [35], 28 showed differential gene or isoform expression in *msh1* S2 mutants (Fig. 2b). Additionally, 95 drought-responsive genes [36] were differentially expressed in *msh1* mutants, with 7 differentially expressed as early as the *msh1* S1 generation (Additional file 7: Figure S3A). Differential expression of drought-responsive genes here is consistent with findings of heat and drought tolerance from *MSH1* perturbation [19]. However, 22 cold-responsive transcription factors [37] were also differentially expressed in *msh1* mutants, with 3 (*STZ*, *ERF2*, *ERF6*) up-regulated in the *msh1* S1 generation (Additional file 7: Figure S3B). The observed changes across multiple abiotic stress pathways indicate that loss of *MSH1* elicits a general stress response, rather than targeted changes to a specific stress. Triggering of these stress pathways in *msh1* mutants likely confers the previously observed stress tolerances, but may also be linked to growth rate.

**Fig. 3:**
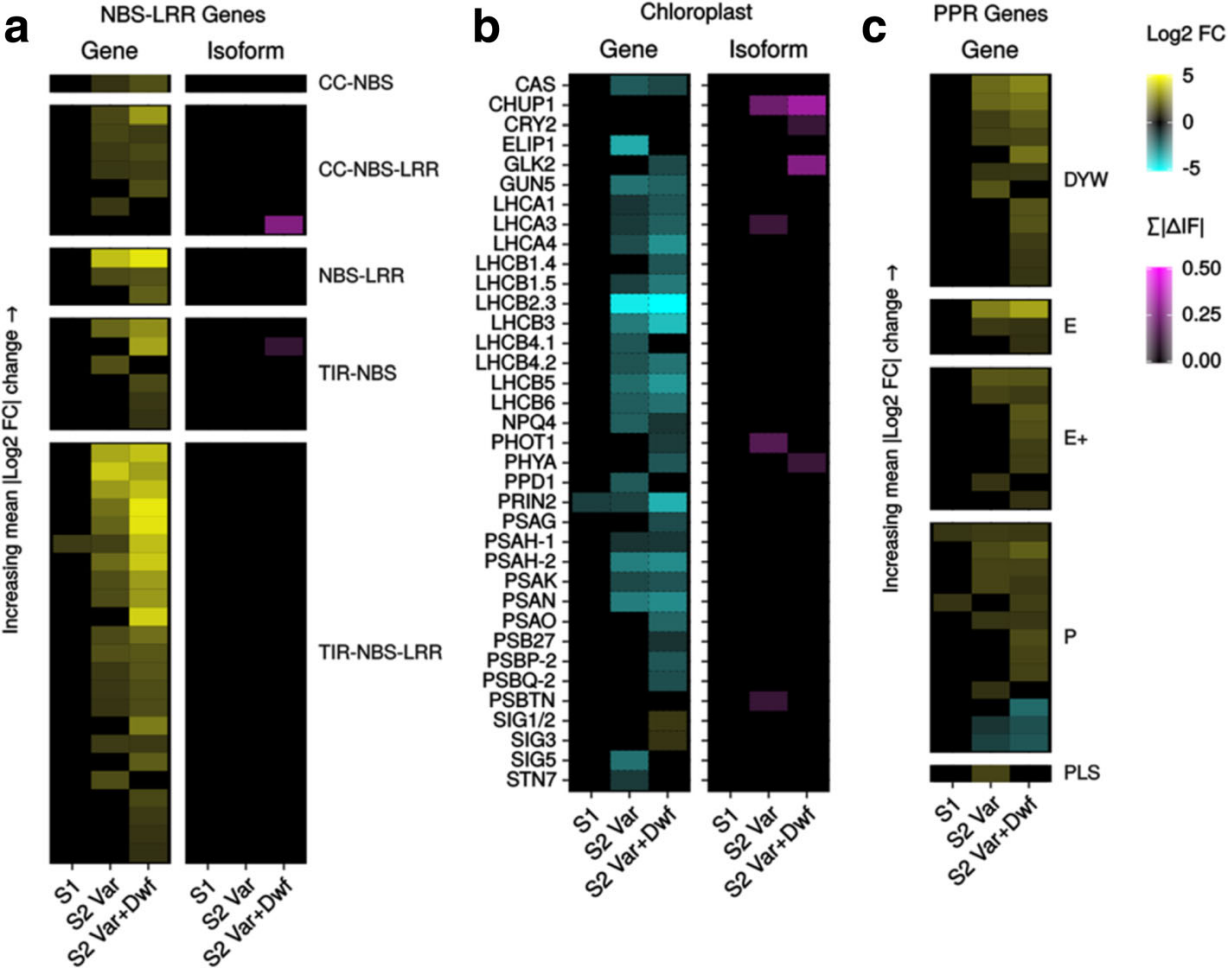
*msh1* mutants show triggering of defense responses and changes to plastid and PPR genes. Changes in expression of transcripts and isoforms in **a**: NBS-LRR genes, **b**: genes essential for photosynthesis and plastid function, and **c**: PPR genes (no alternatively spliced PPR genes were detected)

In addition to abiotic stress, the repeated observation of enriched GO categories related to biotic stress, such as *systemic acquired resistance*, *regulation of defense response*, and *regulation of plant-type hypersensitive response* is likely related to accumulating evidence of crosstalk between ROS signaling and defense responses [38–40]. This would be consistent with the enrichment of *respiratory burst involved in defense response* as early as the S1 generation, and sufficient activation of the immune response triggers a decrease in plant growth [41]. As suggested from enrichment analysis, we found that many genes involved in salicylic acid and jasmonate pathways were differentially expressed (Additional file 8: Figure S4A-B), as well as other phytohormones such as auxin (Additional file 8: Figure S4C). Further inspection revealed that a significant number of *NBS-LRR* genes, the largest family of disease resistance (*R*) genes, were also up-regulated (Fig. 3a). *R* genes can be triggered by a variety of environmental changes [42], and may also be particularly labile to changes in DNA methylation [43].

**Fig. 4:**
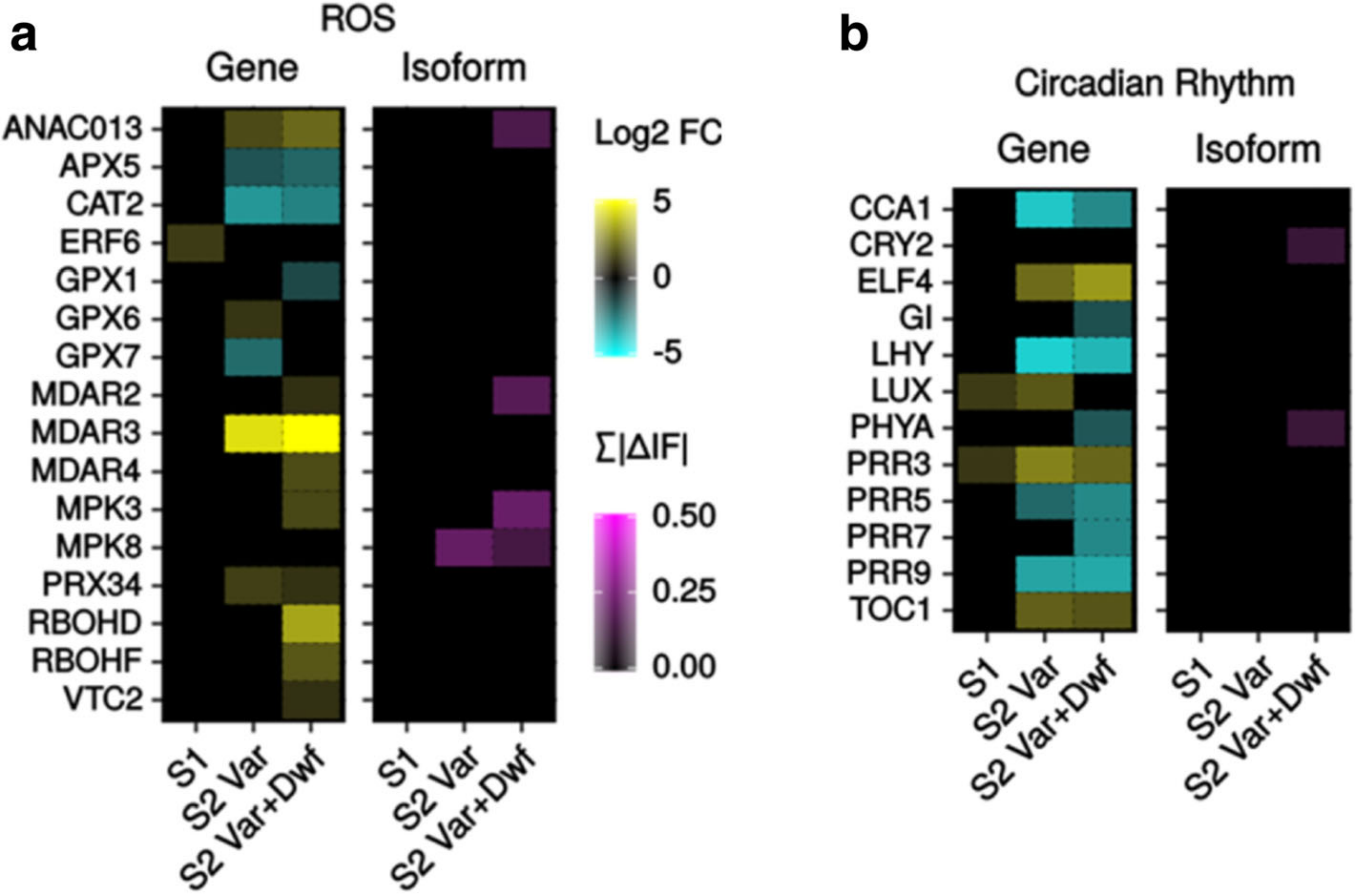
ROS and circadian rhythm genes are altered in *msh1* mutants. Changes in expression of transcripts and isoforms in **a**: ROS related genes, and **b**: circadian rhythm genes

As expected, we also found down-regulation of many genes related to photosynthesis and plastid function, such as light harvesting complex and photosystem I and II components, as well as up-regulation of several *PPR* genes of various families (Fig. 3b-c). This includes down-regulation of *PRIN2*, which is involved in redox-mediated retrograde signaling and expression of genes transcribed by the plastid-encoded RNA polymerase [44], beginning in the *msh1* S1 generation. *GUN5*, the ChlH subunit of Mg-chelatase involved in the tetrapyrrole-based retrograde signaling [45], was also down-regulated in *msh1* -/- S2 plants. Previous studies found altered redox levels in *msh1* mutants, with plastoquinone and phylloquinone biased towards a reduced state [15]. Here we also see differential expression of genes related to redox, including the H_2_O_2_ scavenger *APX5*, and *MDAR3*, involved in the regeneration of reduced ascorbate (Fig. 4a). Among nuclear-encoded plastid or mitochondria-associated genes [46], *msh1* S2 variegated plants had a bias towards down-regulation, while in *msh1* S2 variegated & dwarf plants these genes are more evenly split between up-regulation and down-regulation (Additional file 9: Figure S5A-B). However, 46 and 40% of the same plastid-associated and mitochondrial-associated DEGs, respectively, were shared between the two groups (Additional file 9: Figure S5C-D).

Therefore, while one of the initial responses to *MSH1* loss is transcriptional changes in genes directly associated with the organelles, many other changes occur in the nucleus as an additional consequence. In addition to abiotic and biotic stress pathways previously mentioned, we observed differential expression of several core components of the circadian clock in *msh1* S2 mutants, including *LHY* and *TOC1* (Fig. 4b). In *msh1* S2 variegated & dwarf plants, expression of *GI* was also altered. In addition, *PRR3*, a vascular regulator of *TOC1* [47], is differentially expressed beginning in *msh1* S1 generation mutants. Since circadian rhythm is linked to many other gene networks [48, 49], including ROS and phytohormone pathways that are altered in *msh1* mutants, these changes may be an important contributor to the broad pleiotropy in *msh1* mutants.

Rather than an unrelated assortment of pathways, there is accumulating literature indicating that many of those affected in these *msh1* mutants are interconnected as part of a large signaling network. For example, organelle perturbation is known to affect ROS levels [50], which impacts circadian rhythm and vice versa [49, 51]. In turn, both influence phytohormones [38, 52–54], calcium signaling [55, 56], and biotic and abiotic stress responses [57–60]. Therefore, many of the pathways affected in *msh1* can be considered as a series of direct consequences from the initial organelle perturbation, with signs of this beginning in the *msh1* S1 generation. In support of this, comparison of the *msh1* transcriptome revealed that a number of changes in such pathways were also found in the transcriptomes of plants with other plastid or chloroplast mutations, or chemical disruption of organelle function (Additional file 10: Figure S6) [61–70]. In *msh1* mutants, the number of genes within these pathways that are differentially expressed or spliced, and the intensity of their modulation, are associated with increasing phenotypic severity and comprise a distinguishing feature between *msh1* S1, *msh1* S2 variegated, and *msh1* S2 variegated & dwarf plants.

### Small RNA changes and differential TE expression suggest global chromatin alterations in *msh1* mutants

Because microRNAs are known to cause large effects in gene regulation and phenotype [71], we also performed small RNA-sequencing and aligned 20 to 24-nt reads to the genomic sequences of all high-confidence *A. thaliana* microRNAs annotated by miRBase [72]. In *msh1* S1 plants, only miR156 was up-regulated compared to the wild-type segregant control, and miR156 was also up-regulated in both the *msh1* S2 variegated and *msh1* S2 variegated & dwarf plants (Additional file 11: Table S2). The miR156 regulates the transition from vegetative to reproductive phase, and was previously found to be differentially expressed in *chm1* mutants [14]. miR163, involved in root architecture and secondary metabolites [73, 74], was also found to be up-regulated in both the *msh1* S2 variegated and *msh1* S2 variegated & dwarf plants, as was miR391, whose function is still unclear. miR169, which is involved in stress response and nutrient signaling [75–77], was down-regulated in both the *msh1* S2 variegated and *msh1* S2 variegated & dwarf plants.

**Fig. 5:**
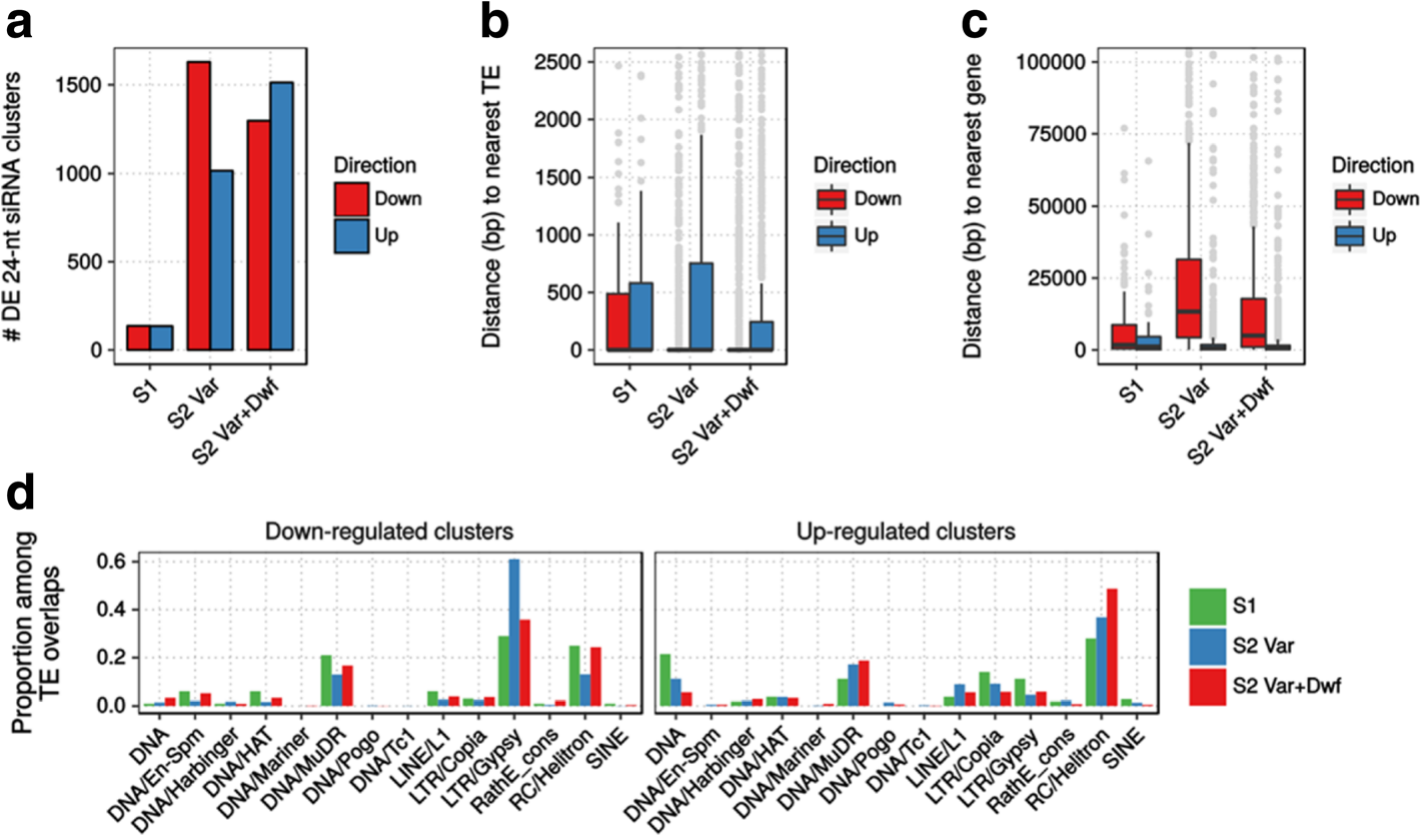
*msh1* mutants have altered 24-nt siRNA levels depending on generation and phenotype. **a** Total number of down-regulated and up-regulated 24-nt siRNA clusters in *msh1* mutants. **b** Boxplot of distance between differentially expressed 24-nt siRNA clusters and the nearest transposable element. **c** Boxplot of distances between differentially expressed 24-nt siRNA clusters and the nearest gene. **d** Distribution of transposable element superfamilies overlapped by differentially expressed 24-nt siRNA clusters, as a proportion within each sample and direction of cluster change

A previous study indicated that the loss of *MSH1* leads to epigenetic changes [20], particularly in phenotypically dwarf plants and with respect to DNA methylation in the CHG and CHH sequence contexts. CHG/CHH methylation is primarily associated with heterochromatic regions and transposable elements, and with short interfering RNA (siRNA) production. Small RNAs are intimately linked with chromatin organization [78], and believed to play a role in some stress responses, both biotic and abiotic [79–81]. We analyzed siRNA levels by mapping small RNA-sequencing reads to the entire genome using ShortStack [82]. Most siRNA clusters identified belonged to the 24-nt class, particularly those that were differentially expressed (Additional file 12: Figure S7A), and so were the focus of further analysis. Analogous to what was observed with gene expression changes, *msh1* S1 plants only had 276 differentially expressed 24-nt siRNA clusters. In contrast, *msh1* S2 variegated and *msh1* S2 variegated & dwarf plants had 2646 and 2810 differentially expressed 24-nt siRNA clusters, respectively. In the *msh1* S2 variegated plants, the majority of these differentially expressed clusters were down-regulated, whereas in the *msh1* S2 variegated & dwarf plants, the majority were up-regulated (Fig. 5a).

In the *msh1* S2 plants, down-regulated 24-nt siRNA clusters are more localized to the pericentromere, whereas up-regulated 24-nt siRNA clusters are more spread throughout the chromosome and encompass euchromatic regions (Additional file 12: Figure S7B). Therefore, down-regulated 24-nt siRNA clusters more frequently overlapped transposable elements (TEs) and were generally closer to TEs than up-regulated 24-nt siRNAs were (Fig. 5b-c, Additional file 12: Figure S7C). However, up-regulated 24-nt siRNAs still tended to be located near TEs, but were also much closer to genes than down-regulated 24-nt siRNAs were. Consistent with this observation, down-regulated 24-nt siRNA clusters were mostly associated with *Gypsy* elements, particularly in *msh1* S2 variegated plants, while up-regulated siRNA clusters were mostly associated with *Helitron* elements, particularly in *msh1* S2 variegated & dwarf plants (Fig. 5d). To a lesser degree, both up-regulated and down-regulated 24-nt siRNA clusters were associated with *MuDR* elements across all *msh1* mutants.

We then evaluated whether any transposable element families showed differential expression in *msh1* mutants using RNA-seq data. *Copia* and *MuDR* elements showed greatest tendency toward differential expression, regardless of direction of change (Additional file 13: Figure S8; Additional file 14: Table S3). Expression of TEs did not appear strongly correlated to siRNA changes, although this may be masked when evaluating at the superfamily or family level rather than individual elements. However, in a similar trend to DEGs and differential siRNA clusters, *msh1* -/- S2 variegated & dwarf plants showed the most changes (71 TE families), followed by *msh1* -/- S2 variegated plants (53 TE families), with *msh1* -/- S1 plants having the fewest (4 TE families). Overall, there were more instances of increased TE expression than decreased TE expression, and two families, *ATHILA4C* and *ATMU8*, had increased expression in all *msh1* mutant groups. Furthermore, in *msh1* S2 variegated and dwarf plants, DEGs were 1.7-fold enriched within 1 kb of *RathE1_cons* elements (chi-square test *p* = 0.003), which were also differentially expressed (Additional file 14: Table S3). Therefore, although not apparently widespread, differential expression of certain transposable elements could be affecting nearby gene expression [21].

We did not find any statistically significant overall enrichment of DEGs proximal to differentially expressed 24-nt siRNA clusters (using maximum distances varying between 50 bp and 10 kb), suggesting that siRNA changes here do not impact gene expression at the genome-wide level in an obvious manner. However, two large gene families, *R* genes and *PPR* genes, tend to be associated with repeats and thus may be susceptible to regulation via siRNAs [83, 84]. Although no enrichment was found for *R* genes, we observed a 2.2-fold enrichment for differential expression of *PPR* genes within 5 kb of a differentially expressed siRNA cluster (chi-square test *p* = 0.02) in *msh1* S2 variegated & dwarf plants, suggesting a possible link between *PPR* gene expression and proximal siRNAs. These findings of differential siRNA and TE expression in *msh1* mutants, coupled with previous studies showing DNA methylation changes, indicate that loss of *msh1* results in broad, global alterations in chromatin organization.

## Discussion

Organelles are essential centers of regulation for multiple cellular signals; seedling, leaf, and flower development; energy and carbon metabolism; and light and temperature adaptation in plants [4, 85–87]. Loss of the dual organelle-targeted gene *MSH1* triggers changes involving plastid and mitochondria states, resulting in altered ROS levels and organellar genome instability, especially within white leaf sectors, and a large reduction in sucrose metabolism [12–15]. The resulting variable phenotypes, which requires two generations of homozygosity for complete elaboration but is not amenable to fixation, complicates studies into its function. Here, we performed RNA-seq and sRNA-seq on mutants newly homozygous for *msh1* T-DNA insertion to expand upon the global and pathway-specific changes associated with *MSH1* loss of function, and in particular to each phenotype.

Using this segregating *msh1* T-DNA material, we found subtle gene expression changes in the first homozogyous generation related to plastid redox regulation, plant defense, temperature response, and circadian rhythm. Despite this, the first homozygous generation shows little alteration in phenotype, and the total number of changes and the magnitude of the changes are still relatively small (196 DEGs). Since *MSH1* expression is particularly high within carpels [88], *MSH1* function may be particularly important during reproduction and the gametophyte stage, where disruption of organelle functions during this stage could have larger consequences on the cell [89]. This would be consistent with the much stronger phenotypes observed by the second sporophytic generation, where gene expression changes expand into additional pathways, including auxin and other phytohormones influencing plant development, along with high induction of stress response pathways such as salt, cold, and oxidative stress.

Consistent with enrichment for abiotic and biotic stress transcriptional changes, *msh1* mutants have been found to be tolerant to high light and heat stress [15, 19]. In plants, repeated stress experience from drought, heat, or pathogens can lead to priming that then facilitates faster future responses within the same generation, and in some cases the underlying mechanism of memory appears to be epigenetic [24, 90]. Whole genome bisulfite-sequencing of *msh1* mutants previously revealed numerous changes in DNA methylation over both gene bodies and transposable elements [20], leading to the possibility of epigenetic feedback as a response to *MSH1* loss and heritable methylation changes at stress-responsive loci [24]. However, identification of causative DNA methylation changes is hampered by spontaneous differences that are a function of generational distance between individual plants or other stochastic events [91, 92]. This has complicated efforts to determine essential locus-specific methylation signals from *MSH1* loss, but the application of new methods in methylome analysis may aid future investigation [93, 94].

**Fig. 6:**
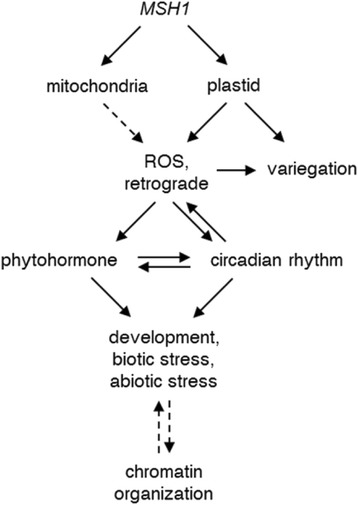
Overview of gene networks altered and other effects resulting from *msh1* loss

As another approach to epigenetic changes, we examined small RNA populations within *msh1* mutants. Much greater 24-nt siRNA changes occurred by the second sporophytic generation of *MSH1* loss than within the first generation, with different preferences in chromosome context (euchromatic or heterochromatic) between up-regulated and down-regulated clusters. However, with the exception of some *PPR* genes, we did not observe an association between differential siRNA levels and gene expression. Thus, although changes in siRNAs, TE expression, and DNA methylation strongly suggest changes to chromatin organization in *msh1* mutants, these could be broad but not locus-specific effects associated with induction of stress responses. Alternatively, the impact of specific chromatin-level changes upon gene expression may simply be uncommon or difficult to conclusively detect, but are important occurrences [95]. A summary schematic of the downstream effects of *MSH1* perturbation, from organelle perturbation to eventual chromatin-level alterations, is depicted in Fig. 6.

Variegation, chlorosis, or stunted growth phenotypes are also observed in several other plastid or chloroplast mutations [96]. In addition, chemical inhibitors of chloroplast biogenesis or function have also been identified. We reanalyzed public data from 12 such mutants or chemical treatments and compared enriched pathways from differentially expressed genes. We found that most mutants or chemical treatments also induced some degree of abiotic and biotic stress pathways that were also enriched in the *msh1* mutant. This supports the broader concept that organelle function is directly linked to stress responses. However, the *MSH1* gene has an unusual combination of characteristics, including a plastid nucleoid localization pattern within the epidermis and vascular parenchyma tissue, and a large number of secondary phenotypes [14, 15, 19]. Furthermore, to our knowledge, no other organellar gene mutant has been directly assayed for changes in DNA methylation [20]. Identification of protein interactors with MSH1 would help place the functional relationship of MSH1 with other chloroplast proteins and variegation mutants. Future studies will also be important for elucidating the direct importance of stress responses, circadian rhythm, and plastid function in *msh1* phenotypes, and could reveal previously undescribed loci under epigenetic regulation.

## Conclusion

T-DNA mutants of the organellar-targeted gene *MSH1* have a wide variety of phenotypes – with variegation and stunted growth being the most obvious among them – but this is only apparent after two generations of homozygosity. Multiple abiotic stress response pathways, pathogen defense, photosynthesis, cell cycle, circadian rhythm, MAPK signaling, and phytohormone regulation are all significantly impacted with far-reaching consequences to development, as the level of their induction differs according with phenotypic severity. Additionally, changes in small RNA populations and transposable element activity provide further evidence of alterations at the chromatin organization level. As *msh1* mutants have been shown to be tolerant to abiotic stress, these results highlight the connection between environment, organelle function, and nuclear regulation, an interplay that is important for understanding stress biology in plants.

## Methods

### Plant growth conditions and RNA extraction

*Arabidopsis* seeds were sown into pots containing Fafard Germination Mix soil added with Turface MVP. After 2–3 days of cold stratification at 4 ° C, pots were moved to growing conditions set at 22 ° C. Light regimes were set on a 12:12 h light–dark cycle with a light intensity of 150 μE m^−2^ s^−1^.

For RNA-seq, individual *msh1* -/- S1, *msh1* -/- S2 variegated, *msh1* -/- S2 variegated & dwarf, and *MSH1* +/+ wild-type segregant plants were used as biological replicates, with 3 replicates per group. Leaf tissue was harvested from plants near bolting developmental phase. For each sample, approximately 100 mg of frozen tissue was ground and extracted for total RNA using a standard TRIzol reagent protocol. RNA samples were then treated with DNaseI (Qiagen catalog #79254).

### Seed stock and PCR genotyping

The *msh1* T-DNA seeds used in this study are part of the Syngenta Arabidopsis Insertion Library (SAIL) collection and ordered from the Arabidopsis Biological Resource Center (germplasm name SAIL_877_F01, stock number CS877617). Plants were genotyped using the following primers:

- LP: 5′ – ACGGAAAAAGTTCTTTCCAGG – 3′
- RP: 5′ – GCTTTCCATCGGCTAGGTTAG – 3′
- LB3: 5′ – TAGCATCTGAATTTCATAACCAATCTCGAT ACAC – 3′

with LP + RP to amplify the wild-type allele and LB3 + RP to amplify the T-DNA insertion. Polymerase chain reaction (PCR) based genotyping was performed using a temperature protocol of 95 ° C for 3 min, followed by 41 cycles of 95 ° C for 30 s, 50 ° C for 30 s, 72 ° C for 90 s, and finally 72 ° C for 5 min. Leaf area was measured using a LemnaTec Scanalyzer HTS.

### RNA-sequencing and analysis

Qiagen RNeasy Plant Mini Kit (Qiagen catalog #74904) was used to clean total RNA samples from *msh1* T-DNA and wild-type segregants prior to RNA-sequencing (RNA-seq). Poly(A)-enriched RNA-seq was performed by CoFactor Genomics, generating at least 30.9 M single-end 50 bp reads per sample. To reduce false positives, we used two software pipelines and only retained concordant results. In both cases, reads were aligned to the Arabidopsis TAIR10 reference genome sequence with annotation from Araport11 PreRelease3.

In the first approach, alignment was performed using TopHat 2.1 (options *--b2-very-sensitive*, default mismatch and edit distance parameters) [97], resulting in ≥30.2 M mapped reads per sample. Cufflinks was used for pre-processing and masking reads derived from rRNAs, tRNAs, organellar-encoded transcripts, and transposable elements. Cuffdiff2 (options *--dispersion-method per-condition --library-norm-method geometric -u -b*) was then used to quantify and detect differentially expressed protein-coding genes (FDR < 0.05, |log2(fold change)| ≥1, and mean FPKM of control or test group ≥1). SpliceR was applied to the Cuffdiff2 output to quantify and characterize differentially expressed isoforms (FDR < 0.05, |∆isoform fraction | ≥0.1, and mean FPKM of control or test group ≥1), and predict nonsense-mediated decay (NMD) sensitive transcripts.

In the second approach, alignment was performed using RUM 2.0.4 (default parameters) [98] keeping only uniquely mapped reads. DESeq2 [99] was used for gene count normalization and to identify differentially expressed genes (FDR < 0.05, |log2(fold change)| ≥1, and mean FPKM of control or test group ≥1). JunctionSeq was used for count normalization of known exons and splice junctions from Araport11 annotation and to then identify genes with at least one differentially used exon or splice junction (gene-wise FDR < 0.05, at least one exon or splice junction with |log2(fold-change)| ≥0.5).

For subsequent gene-level analysis, only those genes which were detected as differentially expressed in the same direction by both Cuffdiff2 and DESeq2 were kept (reported log2(fold-change) values in Results are from Cuffdiff2 estimates). Principle component analysis was based on FPKM (fragments per kilobase million) estimates from Cufflinks, using the 500 most variable genes across samples. For isoform-level analysis, only known isoform variants detected as differentially expressed by Cuffdiff2/spliceR and corresponding to genes with at least one differentially used exon or known splice junction according to JunctionSeq were kept (Additional file 5: Figure S2A-C), reported as the sum of absolute isoform fraction changes from Cuffdiff2/spliceR estimates, or ∑|*Δ*IF|. Isoforms that are sensitive to nonsense mediated decay were identified by spliceR using default parameters, and are indicated in Additional file 2. All gene ontology enrichment analyses were performed using DAVID 6.8b, using the Benjamini method for multiple testing adjustment of *p*-values.

For TE family expression analysis, reads were aligned using the STAR 2-pass method [100] allowing up to 100 multi-mapped locations (per recommendation of TEtranscripts). Quantification and testing for differential expression of TEs were performed using TEtranscripts [101] with the developer-provided Arabidopsis TE family annotation.

### Small RNA-sequencing and analysis

RNA samples of *msh1* T-DNA and wild-type segregants were extracted from the same plants and tissue used in RNA-seq. Small RNA-sequencing (sRNA-seq) was then performed by CoFactor Genomics, generating at least 11.1 M single-end 49 bp reads per sample, which were subsequently trimmed to remove adapters. One replicate of *Msh1* +/+ wild-type segregant was dropped from analysis due to outlier concerns.

ShortStack v3.4 was used to align 20–24 nt sRNA-seq reads to the TAIR10 reference genome sequence with 0 mismatches allowed, resulting in ≥6.7 M mapped reads per sample. ShortStack-identified clusters were combined across all samples (stratified by size class), further merging any clusters within 75 bp of each other and removing those corresponding to miRNA, tRNA, or rRNA loci. This yielded a total of 44,348 clusters across the nuclear genome, with a median cluster length of 208 bp. For each sample, sRNA counts within each cluster by size class were tabulated. DESeq2 was used to identify differential sRNA clusters (FDR < 0.05, |log2 fold-change| ≥0.5).

For miRNA analysis, 20 to 24-nt sRNA-seq reads were aligned using ShortStack 3.4 to the hairpin precursor sequences of all *A. thaliana* miRNAs from miRBase with 0 mismatches allowed, resulting in ≥161 k mapped reads per sample. After filtering these to only include miRNAs annotated as high confidence by miRBase 21, DESeq2 was used to identify differentially expressed miRNAs (FDR < 0.05, |log2 foldchange| ≥1).

### Microarray analysis

Publicly available microarray data were obtained from NCBI Gene Expression Omnibus and are described in Additional file 15: Table S4. For each data set, normalization and analysis was conducted using the R using the package “limma” [102]. For Agilent arrays, data was normalized using the *backgroundCorrect()* and *normalizeBetweenArrays()* functions. For Affymetrix arrays, data was normalized using the *rma*() function. Each data set was subsequently fit to a linear model and calculated for expression statistics using the empirical Bayes approach provided by “limma”*.* Differentially expressed genes against each study’s included wild-type control were selected using a cutoff of FDR < 0.05 and a |log2 foldchange | ≥0.5. Gene ontology enrichment of differentially expressed genes was performed with DAVID 6.8b, using the Benjamini method for multiple testing adjustment of *p*-values.

## Additional files

Additional file 1: Figure S1. a: Schematic of the T-DNA insertion at the *MSH1* locus. b: Diagram of pedigree relationship between T-DNA materials used in this study. Hemizygous MSH1 T-DNA insertion individuals were self-pollinated to create first-generation homozygous *msh1* -/- individuals (S1) as well as homozygous *MSH1* +/+ wild-type segregants. *msh1* -/- S1 plants were then self-pollinated to generated second-generation homozygous *msh1* -/- plants, which showed a range of phenotypes. c: Phenotypic scoring of 260 plants from two *msh1* -/- S2 lines gave an estimated variegation frequency of roughly 70%. (PDF 573 kb)
Additional file 2: Spreadsheet of differentially expressed genes in *msh1* mutants. Only concordant results (with Cuffdiff2 chosen as output) are included. (XLSX 940 kb)
Additional file 3: Table S1. List of genes with switching (from up-regulated to down-regulated or vice versa) or intensifying trends (≥4-fold change in same direction) between S1 and S2 generations. (PDF 460 kb)
Additional file 4: Spreadsheet of differentially expressed isoforms in *msh1* mutants. Concordant results (with Cuffdiff2 chosen as output) and JunctionSeq results are included. (PDF 632 kb) (XLSX 15417 kb)
Additional file 5: Figure S2. Overlap of genes with differential isoform expression from TopHat2 + Cuffdiff2 and genes with at least one differentially used exon or splice junction from RUM + JunctionSeq2, in a: *msh1* S1 plants, b: *msh1* S2 variegated plants, and c: *msh1* S2 variegated & dwarf plants. d: Alternative splicing events and nonsense-mediated decay among differentially expressed isoforms. Each isoform may contain more than one type of alternative splicing event. e: Proportion of differentially expressed isoforms that are predicted to be sensitive to nonsense-mediated decay. f: Overlap of enriched GO terms from differentially expressed genes in *msh1* mutants, and genes with differentially expressed isoforms in *msh1* S2 variegated and dwarf plants. (PDF 632 kb)
Additional file 6: Spreadsheet of GO enrichment results for differentially expressed genes in *msh1* mutants and differentially expressed isoforms in *msh1* S2 variegated & dwarf plants. (XLSX 32 kb)
Additional file 7: Figure S3. Changes in expression of a: transcripts and isoforms of drought-responsive genes, and b: cold-responsive transcription factors. (PDF 879 kb)
Additional file 8: Figure S4. Changes in expression of transcripts and isoforms in a: salicylic acid, b: jasmonate, and c: auxin. (PDF 553 kb)
Additional file 9: Figure S5. Changes in expression of transcripts and isoforms among a: 2163 plastid-associated genes, and b: 1089 mitochondria-associated genes. Overlap of differentially expressed c: plastid-associated genes, and d: mitochondrial-associated genes, between *msh1* mutants. (PDF 532 kb)
Additional file 10: Figure S6. Comparison of transcriptome changes of several organelle mutants and chemical treatments, against biological processes enriched in *msh1* -/- S2 variegated & dwarf plants. Samples are arranged from left to right according to assay type (microarray or RNA-seq) and number of shared enriched categories with *msh1*. For RNA-seq of public data sets, differentially expressed genes were called using DESeq2. To reduce the otherwise very large number of GO enriched categories, only mappings directly annotated by the source database were used (“GO DIRECT”). Only mutants or treatments with at least 4 enriched GO DIRECT categories in common with *msh1* kept retained for analysis. Data were obtained from sources and studies listed in Additional file 7. (PDF 697 kb)
Additional file 11: Table S2. List of differentially expressed miRNA in *msh1* mutants. (PDF 470 kb)
Additional file 12: Figure S7. a: Total number of differentially-expressed siRNA clusters for each size class, by sample. b: Genomic distribution of differentially expressed 24-nt siRNA clusters. c: Proportion of differentially expressed 24-nt siRNA clusters overlapping genes and transposable elements. (PDF 616 kb)
Additional file 13: Figure S8. Number of differentially expressed transposable element families in *msh1* mutants, grouped by superfamily. (PDF 374 kb)
Additional file 14: Table S3. List of differentially expressed TE families in *msh1* mutants. (PDF 491 kb)
Additional file 15: Table S4. List of plastid or chloroplast mutants or chemical treatments used for comparative analysis, and their sources. (PDF 475 kb)

## Abbreviations

DEGs: Differentially expressed gene
FPKM: Fragments per kilobase million
MSH1: MutS Homolog 1
ROS: Reactive oxygen species
S1: First generation homozygous self-pollinated plants
S2: Second generation homozygous self-pollinated plants
sRNA: Small RNA
TE: Transposable element
