# Supplementary figures and images for: Stress-responsive pathways and small RNA changes distinguish variable developmental phenotypes caused by MSH1 loss
Source: BMC Plant Biol. 2017 Feb 20;17:47. doi: 10.1186/s12870-017-0996-4 (PMC5319189; doi:10.1186/s12870-017-0996-4)

**a**

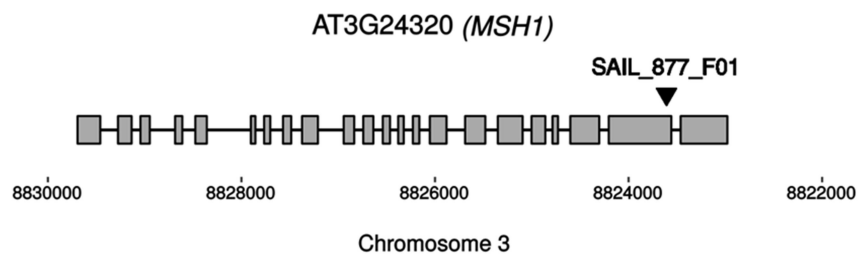

**b**

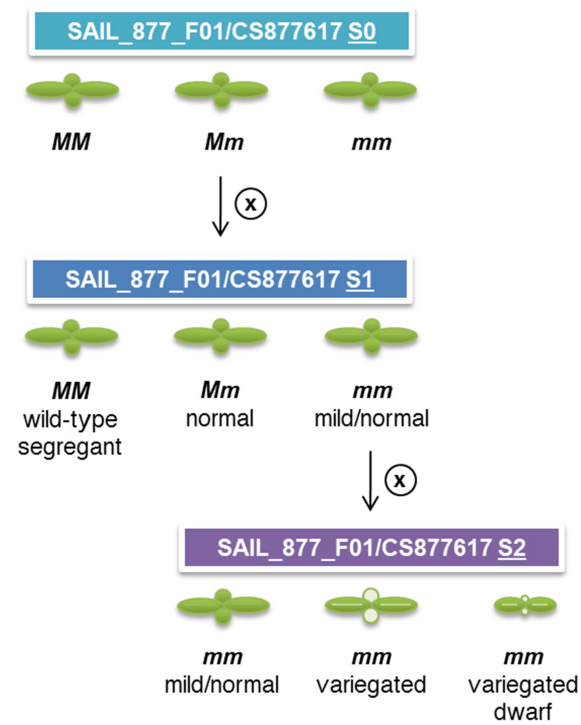

**c**

| Line                        | # plants | # plants with variegation |
|-----------------------------|----------|---------------------------|
| SAIL_877_F01/CS877617 #12-4 | 116      | 77 (66.4%)                |
| SAIL_877_F01/CS877617 #12-9 | 150      | 111 (74.0%)               |

Supplement: Additional file 1: Figure S1. — a: Schematic of the T-DNA insertion at the MSH1 locus. b: Diagram of pedigree relationship between T-DNA materials used in this study. Hemizygous MSH1 T-DNA insertion individuals were self-pollinated to create first-generation homozygous msh1 -/- individuals (S1) as well as homozygous MSH1 +/+ wild-type segregants. msh1 -/- S1 plants were then self-pollinated to generated second-generation homozygous msh1 -/- plants, which showed a range of phenotypes. c: Phenotypic scoring of 260 plants from two msh1 -/- S2 lines gave an estimated variegation frequency of roughly 70%. (PDF 573 kb) [file 12870_2017_996_MOESM1_ESM.pdf]

**a**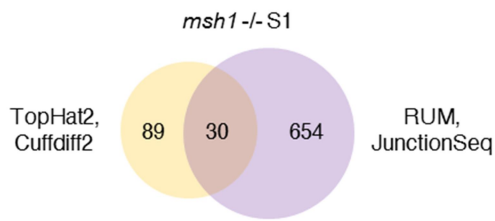**b**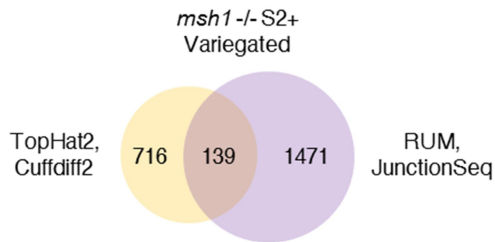**c**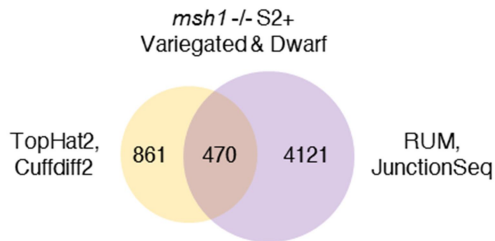**d**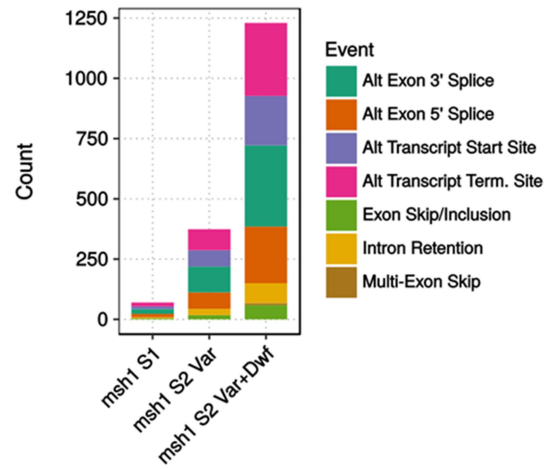**e**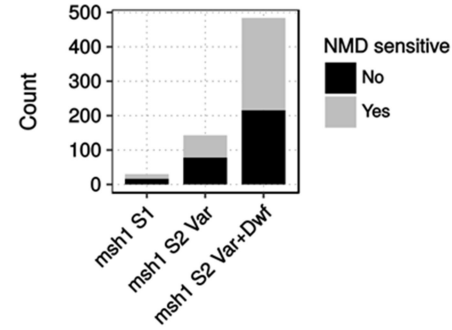**f**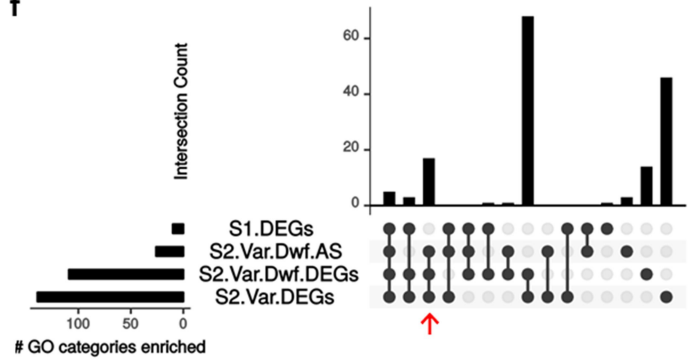

Supplement: Additional file 5: Figure S2. — Overlap of genes with differential isoform expression from TopHat2 + Cuffdiff2 and genes with at least one differentially used exon or splice junction from RUM + JunctionSeq2, in a: msh1 S1 plants, b: msh1 S2 variegated plants, and c: msh1 S2 variegated & dwarf plants. d: Alternative splicing events and nonsense-mediated decay among differentially expressed isoforms. Each isoform may contain more than one type of alternative splicing event. e: Proportion of differentially expressed isoforms that are predicted to be sensitive to nonsense-mediated decay. f: Overlap of enriched GO terms from differentially expressed genes in msh1 mutants, and genes with differentially expressed isoforms in msh1 S2 variegated and dwarf plants. (PDF 632 kb) [file 12870_2017_996_MOESM5_ESM.pdf]

**a**

Drought Responsive

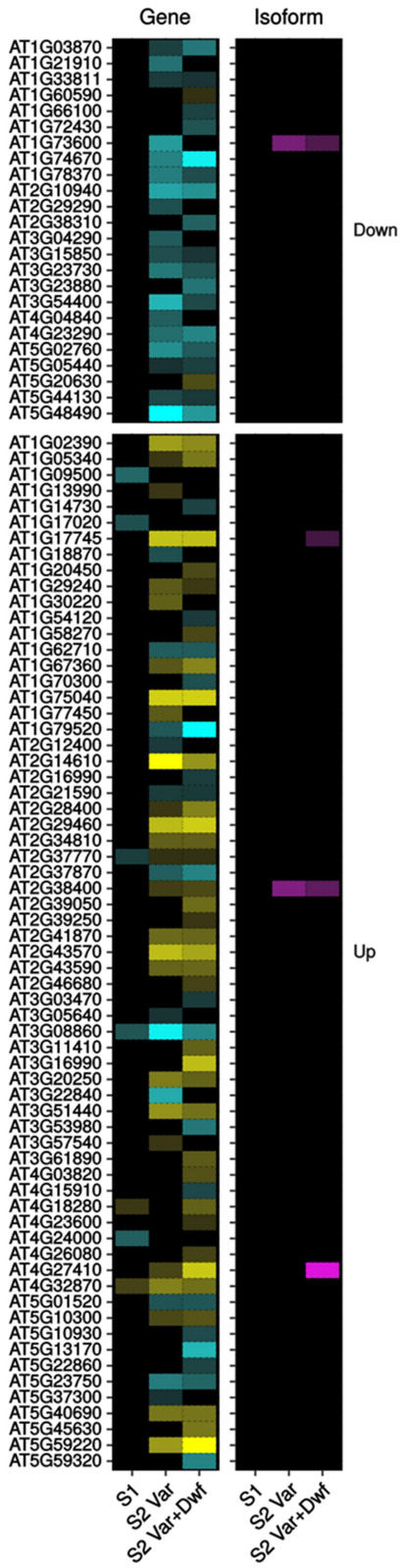**b**

Cold Responsive Transcription Factors

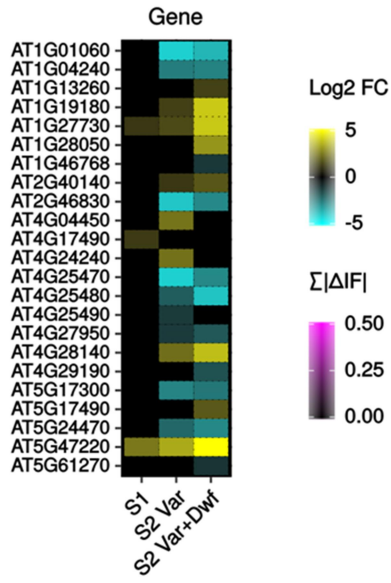

Supplement: Additional file 7: Figure S3. — Changes in expression of a: transcripts and isoforms of drought-responsive genes, and b: cold-responsive transcription factors. (PDF 879 kb) [file 12870_2017_996_MOESM7_ESM.pdf]

**a**

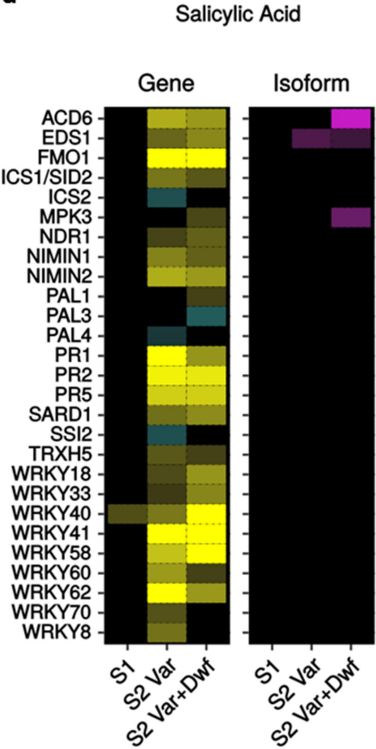

**b**

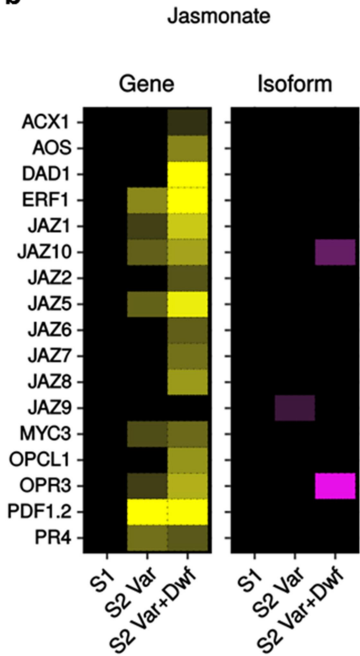

**c**

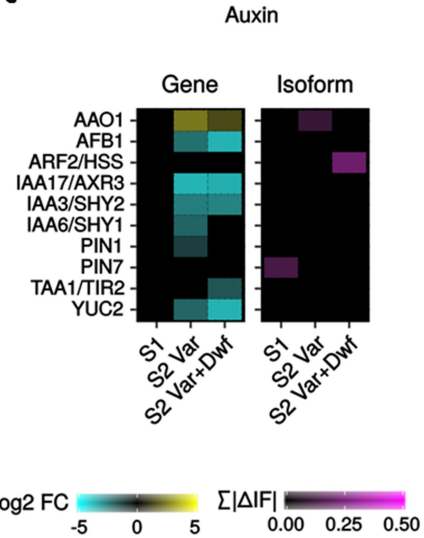

Supplement: Additional file 8: Figure S4. — Changes in expression of transcripts and isoforms in a: salicylic acid, b: jasmonate, and c: auxin. (PDF 553 kb) [file 12870_2017_996_MOESM8_ESM.pdf]

a

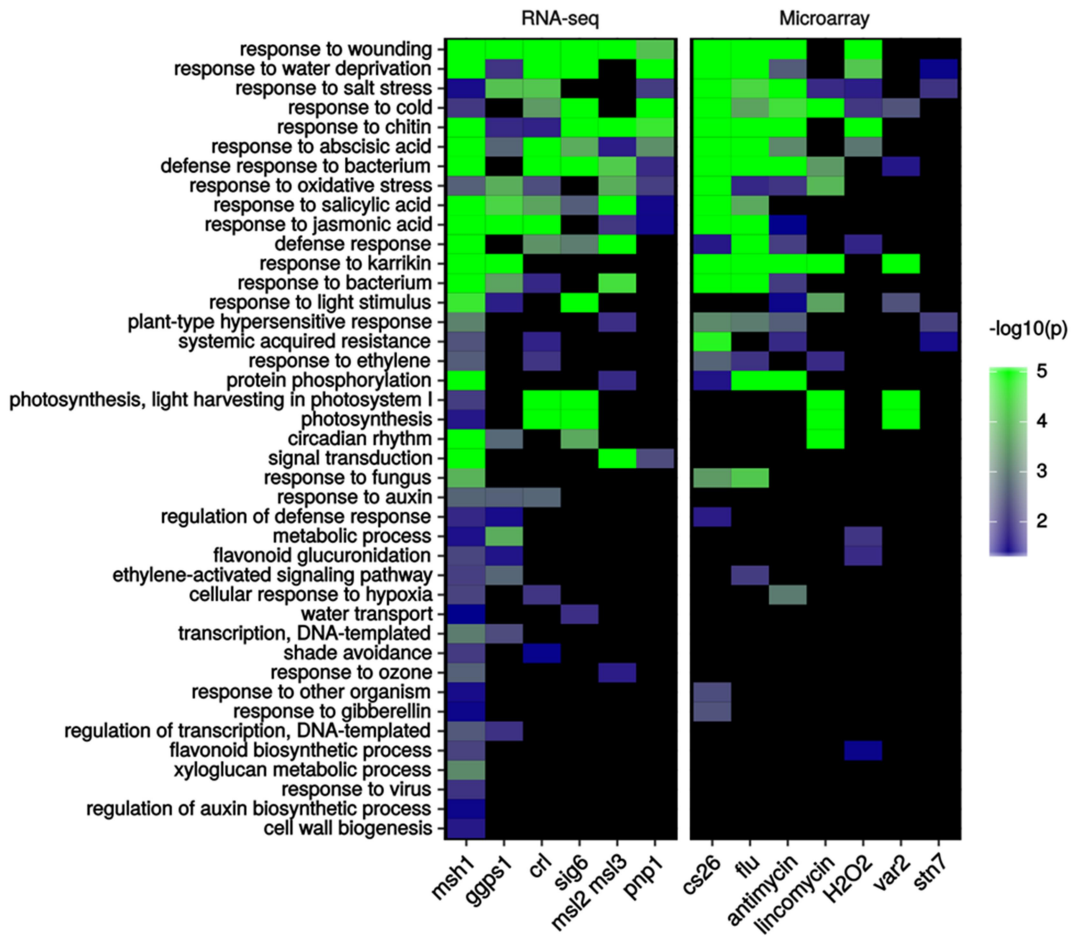

Supplement: Additional file 10: Figure S6. — Comparison of transcriptome changes of several organelle mutants and chemical treatments, against biological processes enriched in msh1 -/- S2 variegated & dwarf plants. Samples are arranged from left to right according to assay type (microarray or RNA-seq) and number of shared enriched categories with msh1. For RNA-seq of public data sets, differentially expressed genes were called using DESeq2. To reduce the otherwise very large number of GO enriched categories, only mappings directly annotated by the source database were used (“GO DIRECT”). Only mutants or treatments with at least 4 enriched GO DIRECT categories in common with msh1 kept retained for analysis. Data were obtained from sources and studies listed in Additional file 7. (PDF 697 kb) [file 12870_2017_996_MOESM10_ESM.pdf]

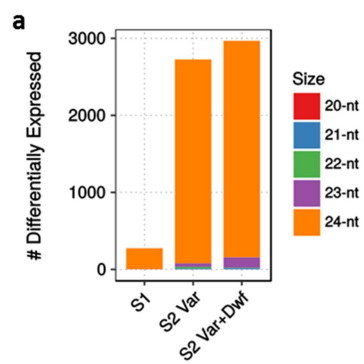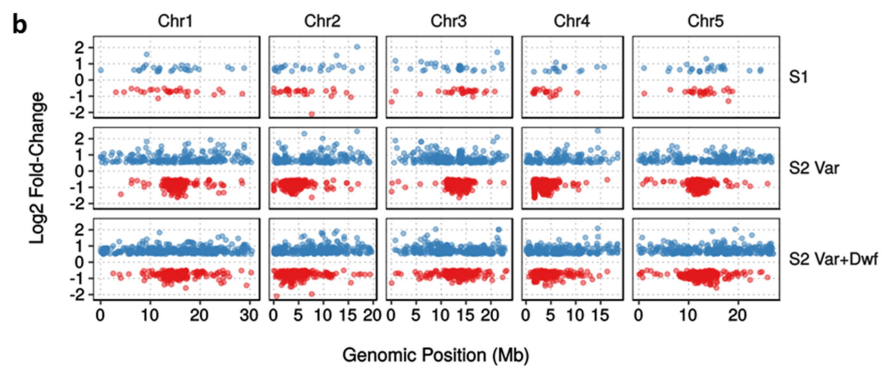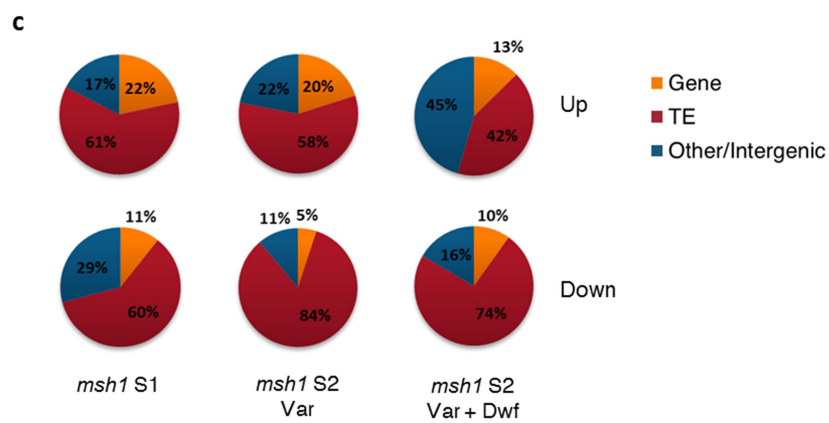

Supplement: Additional file 12: Figure S7. — a: Total number of differentially-expressed siRNA clusters for each size class, by sample. b: Genomic distribution of differentially expressed 24-nt siRNA clusters. c: Proportion of differentially expressed 24-nt siRNA clusters overlapping genes and transposable elements. (PDF 616 kb) [file 12870_2017_996_MOESM12_ESM.pdf]

**a**

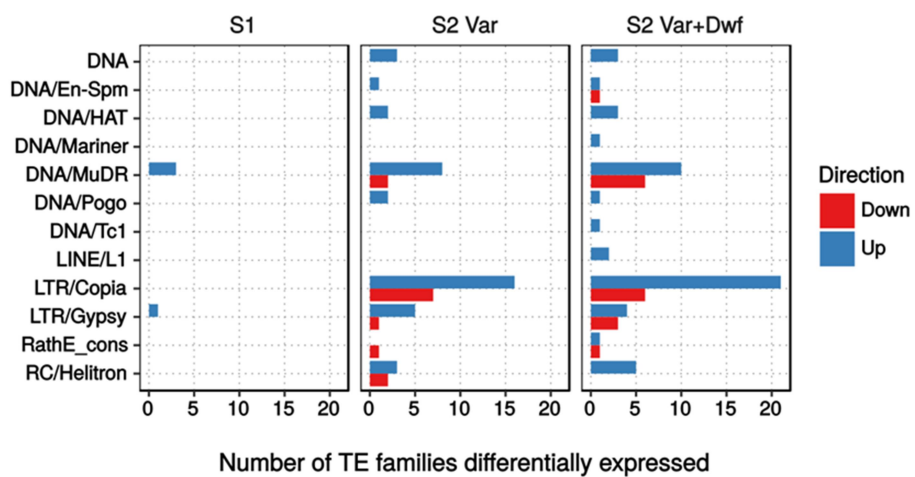

Supplement: Additional file 13: Figure S8. — Number of differentially expressed transposable element families in msh1 mutants, grouped by superfamily. (PDF 374 kb) [file 12870_2017_996_MOESM13_ESM.pdf]
