# Supplementary material for: Stress-responsive pathways and small RNA changes distinguish variable developmental phenotypes caused by MSH1 loss
Source: BMC Plant Biol. 2017 Feb 20;17:47. doi: 10.1186/s12870-017-0996-4 (PMC5319189; doi:10.1186/s12870-017-0996-4)
Supplement: Additional file 3: Table S1. — List of genes with switching (from up-regulated to down-regulated or vice versa) or intensifying trends (≥4-fold change in same direction) between S1 and S2 generations. (PDF 460 kb) [file 12870_2017_996_MOESM3_ESM.pdf]

Table S1: List of genes with switching (from up-regulated to down-regulated or vice versa) or intensifying trends (≥ 4-fold change in same direction) between S1 and S2 generations.

Direction	Gene ID	Name/Description
Switch	*AT1G35140*	Phosphate-responsive 1 family protein (PHI-1/EXL1)
	AT1G32350	alternative oxidase 1D (AOX1D)
	AT2G29350	senescence-associated gene 13 (SAG13)
	AT2G37770	NAD(P)-linked oxidoreductase superfamily protein (AKR4C9)
	AT3G51860	cation exchanger 3 (CAX3)
	AT4G37220	Cold acclimation protein WCOR413 family
	AT4G37990	cinnamyl alcohol dehydrogenase 8
Intensifying	*AT1G10522*	PLASTID REDOX INSENSITIVE 2 (PRIN2)
	AT1G11850	transmembrane protein
	AT1G23710	hypothetical protein (DUF1645)
	AT1G29418	transmembrane protein
	AT1G30720	FAD-binding Berberine family protein
	AT1G56510	Disease resistance protein (TIR-NBS-LRR class) (WRR4)
	AT1G67810	sulfur E2 (SUFE2)
	AT1G72620	alpha/beta-Hydrolases superfamily protein
	AT1G78410	VQ motif-containing protein
	AT1G80840	WRKY DNA-binding protein 40 (WRKY40)
	AT1G27730	salt tolerance zinc finger (STZ)
	AT2G04495	transmembrane protein
	AT2G05380	glycine-rich protein 3 short isoform (GRP3S)
	AT2G14560	LATE UPREGULATED IN RESPONSE TO HYALOPERONOSPORA PARASITICA (LURP1)
	AT2G17040	NAC domain containing protein 36 (NAC036)
	AT2G23340	DREB and EAR motif protein 3 (DEAR3)
	AT2G30020	Protein phosphatase 2C family protein
	AT2G36540	Haloacid dehalogenase-like hydrolase (HAD) superfamily protein
	AT2G35930	plant U-box 23 (PUB23)
	AT2G40330	PYR1-like 6 (PYL6)
	AT2G47130	NAD(P)-binding Rossmann-fold superfamily protein (SDR3)
	AT3G05400	Major facilitator superfamily protein
	AT3G06895	syntaxin KNOLLE-like protein
	AT3G07720	Galactose oxidase/kelch repeat superfamily protein
	AT3G60550	cyclin p3;2 (CYCP3;2)
	AT3G08860	PYRIMIDINE 4 (PYD4)
	AT3G10930	hypothetical protein
	AT3G44260	Polynucleotidyl transferase, ribonuclease H-like superfamily protein (CAF1a)
	AT3G59710	NAD(P)-binding Rossmann-fold superfamily protein
	AT4G13180	NAD(P)-binding Rossmann-fold superfamily protein

AT4G17230	SCARECROW-like 13 (SCL13)
AT4G21870	HSP20-like chaperones superfamily protein
AT4G39260	cold, circadian rhythm, and RNA binding 1 (CCR1)
AT5G22520	hypothetical protein
AT5G27420	carbon/nitrogen insensitive 1 (CNI1)
AT5G05320	FAD/NAD(P)-binding oxidoreductase family protein
AT5G42440	Protein kinase superfamily protein
AT5G47220	ethylene responsive element binding factor 2 (ERF2)
