# Supplementary material for: Stress-responsive pathways and small RNA changes distinguish variable developmental phenotypes caused by MSH1 loss
Source: BMC Plant Biol. 2017 Feb 20;17:47. doi: 10.1186/s12870-017-0996-4 (PMC5319189; doi:10.1186/s12870-017-0996-4)
Supplement: Additional file 9: Figure S5. — Changes in expression of transcripts and isoforms among a: 2163 plastid-associated genes, and b: 1089 mitochondria-associated genes. Overlap of differentially expressed c: plastid-associated genes, and d: mitochondrial-associated genes, between msh1 mutants. (PDF 532 kb) [file 12870_2017_996_MOESM9_ESM.pdf]

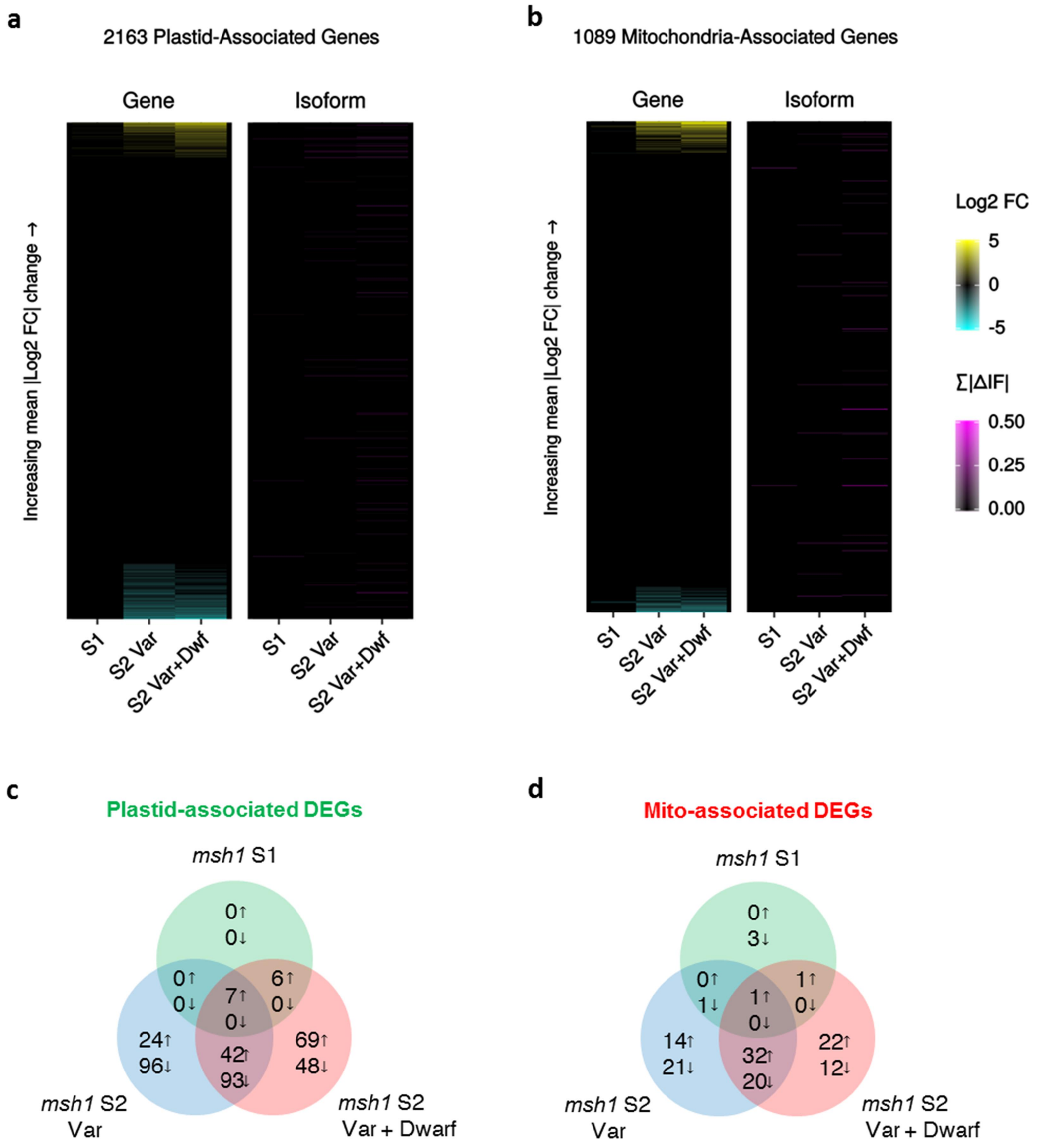
a
2163 Plastid-Associated Genes
Gene
Isoform
Increasing mean |Log2 FC| change →
S1
S2 Var
S2 Var+Dwf
S1
S2 Var
S2 Var+Dwf
b
1089 Mitochondria-Associated Genes
Gene
Isoform
Increasing mean |Log2 FC| change →
S1
S2 Var
S2 Var+Dwf
S1
S2 Var
S2 Var+Dwf
Log2 FC
5
0
-5
Σ|ΔIF|
0.50
0.25
0.00
c
Plastid-associated DEGs
msh1 S1
0↑
0↓
0↑
0↓
6↑
0↓
7↑
0↓
24↑
96↓
42↑
93↓
69↑
48↓
msh1 S2
Var
msh1 S2
Var + Dwarf
d
Mito-associated DEGs
msh1 S1
0↑
3↓
0↑
1↓
1↑
0↓
1↑
0↓
14↑
21↓
32↑
20↓
22↑
12↓
msh1 S2
Var
msh1 S2
Var + Dwarf
