# Supplementary material for: Stress-responsive pathways and small RNA changes distinguish variable developmental phenotypes caused by MSH1 loss
Source: BMC Plant Biol. 2017 Feb 20;17:47. doi: 10.1186/s12870-017-0996-4 (PMC5319189; doi:10.1186/s12870-017-0996-4)
Supplement: Additional file 11: Table S2. — List of differentially expressed miRNA in msh1 mutants. (PDF 470 kb) [file 12870_2017_996_MOESM11_ESM.pdf]

Table S2: List of differentially expressed miRNA in *msh1* mutants.

Sample	Change	miRNA	Isoform	Log2 Fold-Change	FDR
msh1 -/- S1	↑	miR156	*ath-MIR156h*	1.169231	0.024851
msh1 -/- S2 Variegated	↑	miR156	*ath-MIR156h*	1.415798	0.007679
	↑	miR163	*ath-MIR163*	1.346875	6.80E-05
	↓	miR169	*ath-MIR169d*	-2.386886	1.04E-06
			ath-MIR169f	-1.998744	5.52E-06
			ath-MIR169i	-1.321957	0.001224
			ath-MIR169j	-1.854955	1.38E-06
			ath-MIR169k	-1.592382	2.98E-05
			ath-MIR169m	-1.054887	0.000714
			ath-MIR169n	-1.026708	0.007995
	↓	miR170	*ath-MIR170*	-1.228770	7.29E-05
	↓	miR171	*ath-MIR171a*	-1.205889	3.43E-05
	↓	miR319	*ath-MIR319b*	-1.008238	0.023412
	↑	miR391	*ath-MIR391*	1.024847	0.000852
	↑	miR393	*ath-MIR393a*	1.442981	4.55E-07
msh1 -/- S2 Variegated & Dwarf	↑	miR156	*ath-MIR156h*	1.399427	0.008202
	↓	miR157	*ath-MIR157a*	-1.238108	4.66E-06
			ath-MIR157b	-1.231814	4.66E-06
			ath-MIR157c	-1.231421	1.58E-07
			ath-MIR157d	-1.076982	0.001785
	↑	miR163	*ath-MIR163*	1.807864	1.58E-07
	↓	miR164	*ath-MIR164a*	-1.230016	4.55E-07
	↑	miR167	*ath-MIR167c*	1.493322	0.001259
	↓	miR169	*ath-MIR169d*	-1.907430	7.24E-05
			ath-MIR169f	-2.166079	1.13E-06
			ath-MIR169h	-1.210141	0.003284
			ath-MIR169i	-1.798171	2.79E-05
			ath-MIR169j	-2.357833	1.13E-08
			ath-MIR169k	-2.252936	3.83E-08
			ath-MIR169m	-1.990628	1.13E-08
			ath-MIR169n	-1.565536	0.000151
	↑	miR172	*ath-MIR172a*	1.006121	0.001715
	↑	miR391	*ath-MIR391*	1.153562	0.000196
	↓	miR398	*ath-MIR398b*	-1.982932	0.000216
			ath-MIR398c	-1.983621	0.000216
	↑	miR399	*ath-MIR399c*	1.126556	0.000925
	↑	miR837	*ath-MIR837*	1.452763	0.005125
	↑	miR844	*ath-MIR844*	1.289824	0.000158
	↑	miR846	*ath-MIR846*	1.141940	0.000552
	↑	miR5663	*ath-MIR5663*	1.106874	7.24E-05
