# Supplementary material for: Stress-responsive pathways and small RNA changes distinguish variable developmental phenotypes caused by MSH1 loss
Source: BMC Plant Biol. 2017 Feb 20;17:47. doi: 10.1186/s12870-017-0996-4 (PMC5319189; doi:10.1186/s12870-017-0996-4)
Supplement: Additional file 14: Table S3. — List of differentially expressed TE families in msh1 mutants. (PDF 491 kb) [file 12870_2017_996_MOESM14_ESM.pdf]

Table S3: List of differentially expressed TE families in *msh1* mutants.

Sample	Change	Superfamily	Family	Log2 Fold-Change	FDR
msh1 -/- S1	↑	Gypsy:LTR	*ATHILA4C*	1.255310	4.05E-012
	↑	MuDR:DNA	*ARNOLDY1*	1.067460	0.047327
			ATMU8	1.249655	3.16E-006
			VANDAL2	4.301087	2.33E-005
msh1 -/- S2 Variegated	↑↓	Copia:LTR	*ATCOPIA11*	1.520045	1.94E-011
			ATCOPIA12	-1.392682	2.87E-009
			ATCOPIA21	-3.770170	0.022052
			ATCOPIA22	1.281847	1.13E-008
			ATCOPIA27	1.805280	0.000562
			ATCOPIA3	-1.579285	0.000377
			ATCOPIA32	1.414847	0.032776
			ATCOPIA4	-1.165793	0.044068
			ATCOPIA46	-3.039728	5.15E-024
			ATCOPIA5	1.133449	0.000181
			ATCOPIA56	1.168562	0.011978
			ATCOPIA59	1.083888	1.12E-007
			ATCOPIA60	1.051877	0.023800
			ATCOPIA63	-1.963839	0.000008
			ATCOPIA74	-1.363620	0.036450
			ATCOPIA85	1.053532	0.009216
			ATCOPIA88	1.438924	0.022052
			ATCOPIA89	1.266475	0.021803
			ATCOPIA8B	1.271533	0.000570
			ATCOPIA91	1.626531	0.029630
			ATCOPIA95	2.730801	0.005464
			ENDOVIR1	1.549245	7.83E-006
			META1	1.863657	1.16E-008
	↑	DNA	*ATDNA12T3A*	3.818764	4.31E-009
			ATREP19	2.483328	0.010143
			ATTIRX1A	1.645971	0.011096
	↑	En-Spm:DNA	*ATENSPM9*	1.139112	1.65-005
	↑↓	Gypsy:LTR	*ATGP2*	-2.024917	1.12E-007
			ATGP2N	1.270314	6.95E-014
			ATHILA2	2.974612	1.95E-014
			ATHILA4	1.102249	0.007136
			ATHILA4C	1.024619	1.55E-008
			ATHILA4D_LTR	1.494680	0.014303
	↑	HAT:DNA	*ATHATN3*	1.635397	2.86E-011
			SIMPLEHAT2	1.216004	8.25E-011
	↑↓	Helitron:RC	*ATREP11A*	-1.033544	0.000320
			ATREP13	2.246295	8.00E-007

			ATREP2	-1.032147	9.86E-005
			ATREP7	1.236603	0.030644
			HELITRONY1B	1.735326	5.58E-014
	↑↓	MuDR:DNA	*ATDNA2T9C*	2.272841	0.047327
			ATMU11	2.056734	0.041563
			ATMU7	3.122340	0.009216
			ATMU8	1.714242	4.48E-011
			ATREP16	-1.667119	3.46E-009
			BRODYAGA2	1.097068	1.22E-009
			VANDAL11	3.402150	1.66E-006
			VANDAL14	-1.532943	0.023800
			VANDAL1N1	1.147488	4.31E-009
			VANDAL5A	1.349167	0.000299
	↑	Pogo:DNA	*ATHPOGON1*	1.078276	0.044068
			ATHPOGON2	1.535753	0.021883
	↓	RathE2_cons	*RathE2_cons*	-1.282054	0.015532
msh1 -/- S2 Variegated & Dwarf	↑↓	Copia:LTR	*ATCOPIA11*	1.571122	4.05E-012
			ATCOPIA12	-1.593465	1.77E-011
			ATCOPIA22	1.813013	3.74E-016
			ATCOPIA23	1.187184	0.000013
			ATCOPIA27	1.926228	0.000212
			ATCOPIA32	1.517353	0.021837
			ATCOPIA44	-1.220403	0.016617
			ATCOPIA46	-2.913124	1.80E-022
			ATCOPIA49	1.266760	4.12E-007
			ATCOPIA5	1.269765	2.27E-005
			ATCOPIA56	1.223848	0.008327
			ATCOPIA59	1.858569	8.32E-021
			ATCOPIA63	-1.375276	0.001203
			ATCOPIA69	1.155638	3.17E-008
			ATCOPIA70	-1.077221	3.23E-005
			ATCOPIA77	-1.087634	4.24E-006
			ATCOPIA78	1.154896	1.12E-006
			ATCOPIA87	1.124710	3.62E-006
			ATCOPIA88	1.465122	0.020371
			ATCOPIA8B	1.083340	0.003934
			ATCOPIA91	1.716134	0.021837
			ATCOPIA92	1.537597	0.023800
			ATCOPIA93	1.750274	2.99E-008
			ATCOPIA94	1.837094	0.002817
			ATRE1	1.044024	1.40E-006
			ENDOVIR1	1.497001	1.65E-005
			META1	1.917898	4.31E-009
	↑	DNA	*ATDNA12T3A*	4.614003	9.99E-013

		ATTIRX1B	1.130898	0.000366
		ATTIRX1C	1.549097	0.000956
		TNAT1A	1.003719	0.003990
↑↓	En-Spm:DNA	*ATENSPM11*	-1.361081	4.24E-006
		ATENSPM3	2.315965	8.77E-014
↑↓	Gypsy:LTR	*ATGP2*	-1.134568	0.004020
		ATHILA0_I	-1.132122	0.024760
		ATHILA2	2.645276	1.13E-011
		ATHILA4	2.324711	8.48E-010
		ATHILA4A	-1.405585	0.000009
		ATHILA4C	2.715572	4.50E-054
		ATHILA4D_LTR	2.086692	0.000400
↑	HAT:DNA	*ATHAT10*	1.398754	0.001169
		ATHATN10	2.128895	0.000062
		ATHATN3	1.466020	2.85E-009
↑	Helitron:RC	*ATREP13*	1.906370	3.23E-005
		ATREP4	1.295259	3.79E-015
		ATREP7	1.711808	0.002024
		HELITRONY1B	1.430657	6.47E-010
		HELITRONY2	1.031645	4.24E-006
↑	L1:LINE	*ATLINE1_3A*	1.184364	4.12E-009
		ATLINEIII	2.138507	6.53E-034
↑	Mariner:DNA	*DT1*	1.561354	2.20E-005
↑↓	MuDR:DNA	*ARNOLD1*	1.357279	2.25E-010
		ATDNA2T9C	3.755363	0.000704
		ATMU10	1.077571	6.33E-011
		ATMU2	1.045866	1.12E-006
		ATMU4	-4.112173	0.046172
		ATMU8	2.075953	9.37E-016
		ATMUNX1	1.998318	4.86E-007
		ATREP16	-1.189722	2.49E-005
		BRODYAGA2	1.415614	2.78E-015
		VANDAL11	1.948843	0.008670
		VANDAL12	-1.623647	2.61E-012
		VANDAL16	-1.020797	0.012492
		VANDAL17	1.350132	2.31E-017
		VANDAL5A	3.771042	1.68E-026
		VANDAL8	-1.09951	6.44E-010
		VANDAL9	-1.521909	0.023881
↑	Pogo:DNA	*ATHPOGON3*	1.305212	1.66E-006
↑↓	RathE_cons	*RathE1_cons*	1.131339	0.000771
		RathE2_cons	-1.284102	0.015530
↑	Tc1:DNA	*ATTIRTA1*	1.016617	0.016721
