# Supplementary material for: Stress-responsive pathways and small RNA changes distinguish variable developmental phenotypes caused by MSH1 loss
Source: BMC Plant Biol. 2017 Feb 20;17:47. doi: 10.1186/s12870-017-0996-4 (PMC5319189; doi:10.1186/s12870-017-0996-4)
Supplement: Additional file 15: Table S4. — List of plastid or chloroplast mutants or chemical treatments used for comparative analysis, and their sources. (PDF 475 kb) [file 12870_2017_996_MOESM15_ESM.pdf]

Table S4: List of plastid or chloroplast mutants or chemical treatments and their sources used for comparative analysis.

Sample	Type	Tissue	Stage	Light conditions	Gene/Treatment Description	NCBI Accession	Ref
ggps1	RNA-Seq	White leaf sectors	5 weeks	16:8 light-dark	Geranylgeranyl diphosphate synthase involved in isoprenoid synthesis. Mutants have albino leaf sectors.	BioProject PRJNA300851	62
crl	RNA-Seq	Leaf	3 weeks	16:8 light-dark	*CRL* localizes to outer membrane envelope of plastids. Mutants are small with crumpled leaves.	BioProject PRJNA300851	62
msl2 msl3	RNA-Seq	Leaf	3 weeks	16:8 light-dark	*MSL2* and *MSL3* localize to plastid envelope and may function as mechanically-gated ion channels. Mutants are variegated.	BioProject PRJNA300851	62
pnp1	RNA-Seq	Leaf	25 days	16:8 light-dark	Chloroplast polynucleotide phosphorylase important for chloroplast ncRNAs. Mutants are small and paler green.	BioProject PRJNA80171	63
sig6	RNA-Seq	Seedling	2 days	Continuous light	Sigma factor utilized by plastid-encoded RNA polymerase. Mutants have pale green phenotype.	SRA SRP013336	64
var2	Agilent Arabidopsis V4 array	White leaf sectors	4 week old	12:12 light-dark	FtsH2 metalloprotease that functions in thylakoid membrane biogenesis and PSII repair. Mutants are variegated.	GEO GSE18646	65
cs26	Affymetrix ATH1 array	Leaf	3 weeks	16:8 light-dark	Cysteine synthase important for light-dependent redox regulation. Mutants are small and pale under long-day conditions.	GEO GSE19241	66
flu	Affymetrix ATH1 array	Leaf	3 weeks	3 weeks continuous light, then 8 hr dark, then shifted to light and harvested after 2 hr.	Localizes to chloroplast membrane and is important for chlorophyll biosynthesis. Mutants release singlet oxygen upon dark-light shift, causing bleaching and death.	GEO GSE10812	67
stn7	Affymetrix ATH1 array	Leaf	23 day old	20 days continuous light, PSI-optimized light for 3 days, then shifted to PSII-optimized light and harvested after 1 hr.	Thylakoid protein kinase important for redox state transitions. Under shifts between PSI-PSII light, mutant has reduced growth.	GEO GSE42710	68
**H_2O_2**	Affymetrix ATH1 array	Seedling	14 day old	16:8 light-dark	Treated with 20 mM H_2O_2 for 3 hr.	GEO GSE41136	69
**Antimycin A**	Affymetrix ATH1 array	Seedling	14 day old	16:8 light-dark	Sprayed with 50 µM antimycin a, then harvested after 3 hr. Antimycin A is an electron transport inhibitor.	GEO GSE36011	70
**Lincomycin**	Affymetrix ATH1 array	Seedling	5 day old	-	Germinated on media containing 220 µg/ml lincomycin, which inhibits chloroplast biogenesis.	GEO GSE5770	71
